# Proteomic Signature of the Murine Intervertebral Disc

**DOI:** 10.1371/journal.pone.0117807

**Published:** 2015-02-17

**Authors:** Matthew R. McCann, Priya Patel, Agya Frimpong, Yizhi Xiao, Walter L. Siqueira, Cheryle A. Séguin

**Affiliations:** 1 Department of Physiology and Pharmacology, Schulich School of Medicine & Dentistry, The University of Western, Ontario, London, Ontario, N6A 5C1, Canada; 2 School of Dentistry and Department of Biochemistry, Schulich School of Medicine & Dentistry, The University of Western, Ontario, London, Ontario, N6A 5C1, Canada; National Centre for Scientific Research, ‘Demokritos’, GREECE

## Abstract

Low back pain is the most common musculoskeletal problem and the single most common cause of disability, often attributed to degeneration of the intervertebral disc. Lack of effective treatment is directly related to our limited understanding of the pathways responsible for maintaining disc health. While transcriptional analysis has permitted initial insights into the biology of the intervertebral disc, complete proteomic characterization is required. We therefore employed liquid chromatography electrospray ionization tandem mass spectrometry (LC-ESI-MS/MS) protein/peptide separation and mass spectrometric analyses to characterize the protein content of intervertebral discs from skeletally mature wild-type mice. A total of 1360 proteins were identified and categorized using PANTHER. Identified proteins were primarily intracellular/plasma membrane (35%), organelle (30%), macromolecular complex (10%), extracellular region (9%). Molecular function categorization resulted in three distinct categories: catalytic activity (33%), binding (molecule interactions) (29%), and structural activity (13%). To validate our list, we confirmed the presence of 14 of 20 previously identified IVD-associated markers, including matrix proteins, transcriptional regulators, and secreted proteins. Immunohistochemical analysis confirmed distinct localization patterns of select protein with the intervertebral disc. Characterization of the protein composition of healthy intervertebral disc tissue is an important first step in identifying cellular processes and pathways disrupted during aging or disease progression.

## Introduction

Non-specific low back pain has become one of the most common health problems worldwide, with recent reports indicating a lifetime prevalence as high as 84% [1]. According to the most recent Global Burden of Disease study [2] back pain is the single most common cause of disability, with a global prevalence of 23%, causing chronic disability in approximately 12% of the population [1]. Alarmingly, while the prevalence of low back pain has increased over the past three decades [3], current treatment options do not adequately provide improved outcomes [4]. Even though the etiology of low back pain remains unknown, the first manifestations are thought to be a result of degeneration of the intervertebral disc (IVD).

The IVD is a specialized connective tissue structure located between the vertebrae of the spine, permitting flexion, extension, lateral bending, and axial rotation. The IVD consists of three interdependent tissues, the cartilage endplates (CEP), annulus fibrosus (AF) and nucleus pulposus (NP)[5]. During aging and degeneration the IVD undergoes substantial changes in tissue composition [6]. Biochemically, the CEP undergoes calcification [7,8] thereby impeding nutrient flow to the IVD [7]. The elastin content increases in the AF [9], and increased proteoglycan degradation in the NP leads to decreased disc height and an inability of the IVD to maintain turgor against compressive loading [10].

Recent studies have reported the transcriptional profile of IVD tissues in various animal models including rat [11], bovine [12], canine [13] and rabbit [14]. In addition, a small panel of transcripts [15] and microRNA [16] have been associated with IVD tissue in humans, and novel genetic variants have been associated with pathological lumbar disc degeneration [17]. To date however, there are limited studies using unbiased proteomic strategies to define the composition of the healthy IVD. Recent studies provided detailed compositional analysis of the extracellular matrix of multiple human cartilaginous tissues, including the IVD [18] as well as bone and cartilage tissue in zebrafish [19]. These studies highlight the value of global proteomic analysis in establishing the composition of specialized skeletal tissues, and the need to expand this analysis to model organisms commonly used to study development, aging or disease states.

Liquid chromatography coupled with mass spectrometry has emerged as an effective tool for quantitative proteomic profiling of complex tissue extracts [20]. In the current study, we employed a mass-spectrometry approach with a label-free method to decipher the proteomic profile of the healthy murine IVD. We aimed to gain a more in-depth understanding of the proteomic signature of the IVD, including intracellular proteins (transcription factors, metabolic enzymes, etc) and secreted molecules (growth factors, cytokines and matrix components) that regulate the complex microenvironment of IVD. This characterization enables a basic understanding of IVD biology which may ultimately contribute to the identification of targets to modulate IVD health.

## Materials and Methods

### Animals

Animal care and handling procedures in this study were approved by the Animal Use Committee of the University of Western Ontario (AUP 2009–050) in accordance with the guidelines of the Canadian Council on Animal. Fourteen-week-old (skeletally mature) male CD1 mice [21] were sacrificed by CO_2_ asphyxiation. Spinal columns were dissected and cleaned of surrounding tissue.

### Intervertebral Disc Harvest, Protein Extraction and Tryptic Digestion

Intact IVDs (including NP, AF and CEP) were isolated from the thoracic regions by microdissection by shearing the IVD from the adjacent vertebral bone. IVDs were placed in phosphate buffered saline (PBS) under a stereoscope and surrounding muscle, bone, and spinal cord tissues were removed. Four to five thoracic IVDs from each mouse were minced with a scalpel, transferred to a microcentrifuge tube and incubated in 4 M urea, 10 mM dithiothreitol and 50 mM NH_4_HCO_3_ (pH 8.6), for 2 h at room temperature with gentle agitation. Samples were centrifuged at 3,000 *g* for 3 min, the supernatant was collected and total protein concentration was assessed using the Micro Bicinchoninic Acid (BCA) assay (Pierce, Rockford, IL). Equivalent amounts of protein (10 μg) were subjected to tryptic digest (2% trypsin per weight of protein in 50 mM NH_4_HCO_3_, pH 7.8) for 18 h at 37°C.

### Liquid Chromatography Electrospray Ionization Tandem Mass Spectrometry (LC-ESI-MS/MS)

Peptide separation and mass spectrometric analyses were carried out with a nano-HPLC Proxeon (Thermo Scientific, San Jose, CA) linked to a mass spectrometer (LTQ-Velos, Thermo Scientific) using electrospray ionization in a survey scan in the range of m/z values 390–2000 tandem MS/MS [22]. Samples were resuspended in 20 μl of 97.5% H_2_O/2.4% acetonitrile/0.1% formic acid and then subjected to reversed-phase LC-ESI-MS/MS. The nano-flow reversed-phase HPLC was developed with linear 80 min gradient ranging from 5% to 55% of solvent B in 65 min (97.5% acetonitrile, 0.1% formic acid) at a flow rate of 300 nl/min with a maximum pressure of 280 bar. Electrospray voltage and the temperature of the ion transfer capillary were 1.8 kV and 250°C respectively. Each survey scan (MS) was followed by automated sequential selection of seven peptides for CID, with dynamic exclusion of the previously selected ions.

The resulting MS/MS spectra were searched against mouse databases (Swiss Prot and TrEMBL, Swiss Institute of Bioinformatics, Geneva, Switzerland, http://ca.expasy.org/sprot/) using the SEQUEST algorithm in the Proteome Discoverer 1.3 software (Thermo Scientific, San Jose, CA, USA). Search results were filtered for a false discovery rate of 1% employing a decoy search strategy utilizing a reverse database. An additional inclusion criterion for positive identification of proteins was the presence of at least 2 different peptides from a protein and the same protein passing the filter score from at least in four different MS analyses from a total of six MS analyses [23,24].

### Bioinformatic Analysis

Pathway analysis, molecular function, biological processes and cellular components of proteins were obtained from GeneOntology and charts were created using the PANTHER (Protein ANalysis Through Evolutionary Relationships) (http://pantherdb.org; version 9.0) classification system [25]. The PANTHER database allows for high throughput functional analysis of large datasets of protein sequences.

### Validation using Previously Published IVD Markers

To validate the accuracy of the identified proteins, we specifically queried for the presence of 20 IVD-associated markers (proteins or genes) that were previously reported to be expressed in the murine embryonic node [26], developing murine IVD [27], or human IVD [28]. Candidate markers were selected to include a range of protein classifications including extracellular matrix proteins, transcriptional regulators, and secreted proteins representing all tissue types of the IVD.

**Histology and Immunohistochemistry**. To minimize biological variability, immunohistochemical validation was conducted on spine tissue from the same mice used for proteomic analysis. Lumbar spines were fixed in 4% paraformaldehyde overnight at 4°C, washed in PBS, decalcified for 5 days at room temperature in Shandon’s TBD-2 (Fisher Scientific), dehydrated in a graded series of ethanol, cleared in xylene and embedded in paraffin. Samples were sectioned sagittally at a thickness of 5 μm using a microtome (Leica Microsystems, Concord, ON). Sections were then de-waxed in xylene and rehydrated by successive immersion in descending concentrations of alcohol. Serial sections were processed with 0.1% Safranin-O/0.02% fast green to detect sulphated glycosaminoglycans and imaged on a Leica DM1000 microscope with Leica Application Suite. Serial sections were processed for immunohistochemistry following antigen retrieval with 10 mM sodium citrate for 20 min at 95°C, with the exception of sections processed for the localization of Sox9, where 1% Trition-X in PBS was used for 20 min at room temperature. Slides were then blocked with 5% donkey serum in PBS with 0.2% Tween-20 (PBST) (Sigma), for 1 h and then incubated with primary antibody directed against NFAT5 (1:50; Santa Cruz, SC-5499), versican (1:100; raised to the N-terminal 13-residue peptide sequence of human versican [29]), Sox9 (1:200; Santa Cruz, SC17340) or BSP (1:250, Renny Franceschi, University of Michigan, Ann Arbor, USA [30]) in a humidified chamber overnight at 4°C. Slides were washed with PBST and incubated with species-specific secondary antibody (1:200; Alexa Fluor 488, Life Technologies) for 60 min prior to mounting with VECTASHIELD Medium with DAPI (Vector Laboratories, Burlingame, CA). Images were captured with a Zeiss Axio Imager.M1 fluorescence microscope and processed with Northern Eclipse (Empix) software.

## Results

SEQUEST identified 1940 proteins in the tryptic digest of IVD tissues from 14-week-old, skeletally mature wild-type mice. Proteins were filtered by accession numbers and queried using the UniProt database to identify peptides that were not annotated within the database at the time of analysis (UniProt release 2013_06) or that were considered putative uncharacterized (580), as well as any proteins that were derived from Ensembl automatic analysis pipeline and were therefore considered preliminary (396 proteins) listed in S1 Table. This initial filter reduced the list to 1360 proteins localized to the IVD, presented in S2 Table.

**Fig 1:**
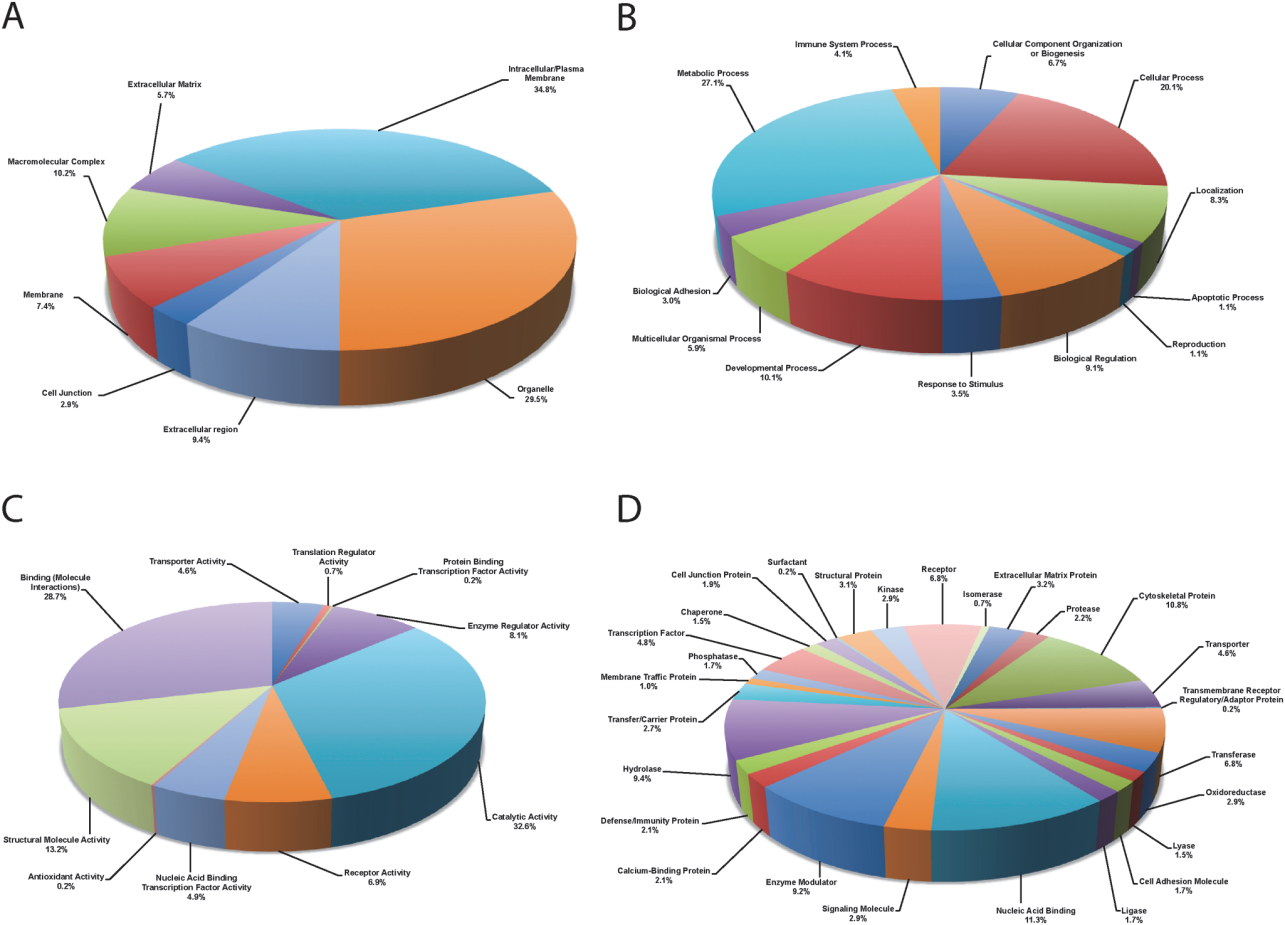
Proteomic signature of the murine intervertebral disc. Pie chart depicting gene ontology (GO) analysis of proteins identified in the murine intervertebral disc. Representation of the distribution of proteins according to their molecular function as **(A)** biological process, **(B)** molecular function, **(C)** cellular component, and **(D)** protein class. Categorizations were based on information provided by the PANTHER classification system (www.pantherdb.org; v9.0). A total of 10 groups for molecular function, 12 groups for biological process, 7 cellular components, and 27 protein classes were detected. Further analysis of the intracellular classification revealed that the majority of proteins were characterized as cytoskeletal, followed by cytoplasmic, organelle and protein-transport ATP synthase complex.

The PANTHER database was then used to classify the list of proteins into categories according to cellular component, molecular function, biological processes and protein class. Based on gene ontology terms, the identified proteins were found to be primarily associated with intracellular/plasma membrane (35%), organelle (30%), macromolecular complex (10%), and extracellular region (9%) (Fig. 1A). The list of total proteins was further classified by biological process into 12 subgroups. The most abundant subgroups were metabolic process (27%), cellular process (20%), and developmental process (10%) (Fig. 1B). Following categorization by molecular function, proteins were mostly grouped into three categories: catalytic activity (33%), binding (molecule interactions) (29%), or structural activity (13%) (Fig. 1C). Characterization based on protein class was also performed, with proteins categorized into 27 classes, with nucleic acid binding (11%) being the most common, followed by cytoskeletal proteins (11%), hydrolases (9%), enzyme modulators (9%) and receptors (7%) (Fig. 1D).

To validate the identified proteins, we queried for candidate IVD-associated genes previously identified based on transcriptional profiling and protein localization studies of the murine and human IVD [26–28,31–33] (Table 1). Of the 20 markers selected, our proteomic approach validated expression of 14 proteins, including the extracellular matrix proteins collagen alpha-1(I), collagen alpha-2(I), and the proteoglycans aggrecan core protein, versican, decorin and biglycan. We also confirmed the presence of both keratin, type I cytoskeletal 8 (cytokeratin 8) and keratin, type I cytoskeletal 19 (cytokeratin 19) within the IVD, established as markers of notochord cells. Members of the Sox family of transcriptional regulators known to be important for cartilage development [34] were screened, with both of Sox5 and Sox9 detected within the IVD. However, the secreted matricellular proteins CCN2 (CTGF) and CCN1 (Cyr61) were not identified. While Tgf-β2 was not identified, Tgf-β3 a related member of the Tgf-β superfamily was detected.

**Table 1:** Validation using previously identified genes and proteins in the murine intervertebral disc.

UniProtKB Accession no.	Gene^†^	Name^†^	Presence/ Absence	Reference
Q61838	A2M	Alpha-2-macroglobulin	+	Minogue *et al*. [28]
Q61282	Acan	Aggrecan core protein	+	Minogue *et al*. [28]
P28653	Bgn	Biglycan	+	Johnstone *et al* [31]
Q6ZQ08	Cnot1	CCR4-NOT transcription complex subunit 1	+	Tamplin *et al*. [26]
P29268	Ccn2	Connective tissue growth factor	-	Bedore *et al*. [32]
P11087	Col1α	Collagen alpha-1(I) chain	+	Minogue *et al*. [28]
Q01149	Col2α	Collagen alpha-2(I) chain	+	Minogue *et al*. [28]
P28654	Dcn	Decorin	+	Johnstone *et al* [31]
Q61221	Hif-1α	Hypoxia-inducible factor 1-alpha	+	Minogue *et al*. [28]
Q3TRM5	Ibsp	Integrin-binding Sialoprotein	-	Minogue *et al*. [28]
P11679	Krt8	Keratin, type I cytoskeletal 8	+	Minogue *et al*. [28]
P19001	Krt19	Keratin, type I cytoskeletal 19	+	Minogue *et al*. [28]
Q14BI5	Myom2	Myomesin 2	+	Tamplin *et al*. [26]
O88942	NFATc1	Nuclear factor of activated T-cells, cytoplasmic 1	-	Sohn *et al*. [27]
P09084	Pax1	Paired box protein Pax-1	-	Sohn *et al*. [27]
P35710	Sox5	Transcription Factor Sox-5	-	Smits *et al*. [42]
Q8BSS6	Sox6	Transcription Factor Sox-6	+	Minogue *et al*. [28]
Q04887	Sox9	Transcription Factor Sox-9	+	Minogue *et al*. [28]
P27090	Tgfβ2	Transforming growth factor beta-2	-	Sohn *et al*. [27]
Q62059	Vcan	Versican core protein	+	Sohn *et al*. [27]

^†^Details obtained from UniProt database (www.uniprot.org/).

**Fig 2:**
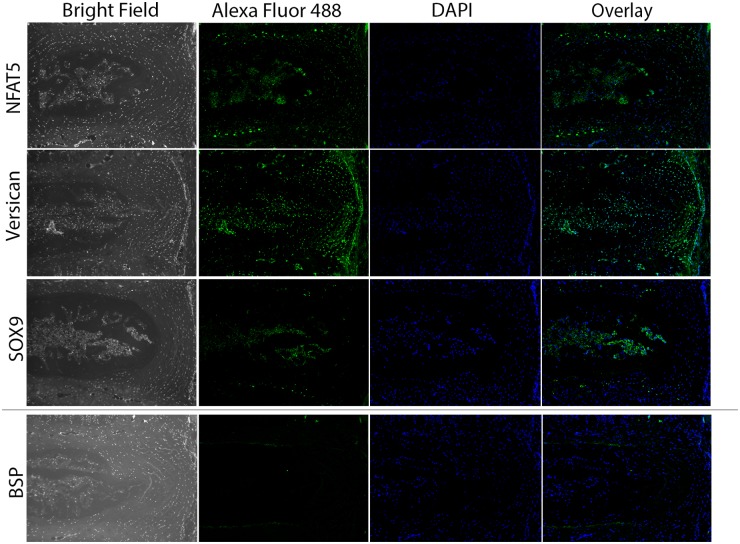
Immunohistochemical staining validating the localization of proteins in the murine intervertebral disc. Representative images demonstrating the immunolocalization of NFAT5, versican, Sox9, and BSP within the murine IVD. Each protein demonstrates a distinct pattern of localization within specific compartments of the disc. NFAT5 is localized to the NP and CEP, versican is present throughout the disc with high levels detected in the outer annulus, Sox9 is present in NP and CEP and BSP is only detected in the CEP. For each protein-specific antibody, sections corresponding to 3 IVDs were assessed for each animal; n = 3 mice. Scale bar = 100 μm.

Immunohistochemical analysis was performed to further confirm expression and tissue-specific localization of a subset of IVD proteins (Fig. 2). NFAT5 was localized to NP and CEP cells, with the expected nuclear and perinuclear subcellular localization. Versican staining was detected throughout the disc with high levels detected in the pericellular matrix of fibrocartilagenous cells of the outer annulus. As expected, Sox9 was detected in NP, CEP, and inner AF but was absent from cells of the outer AF. In contrast to previous reports [12] detection of Bone sialoprotein (BSP) was limited to the CEP, localized to the pericellular matrix.

## Discussion

In this study, we employed a shotgun proteomic approach to determine the proteomic signature of the healthy murine IVD. Our findings provide a comprehensive and efficient resource for understanding the pathways responsible for maintaining disc homeostasis.

To validate the proteomic characterization of the IVD, we employed a candidate approach to specifically query IVD-associated genes previously identified by transcriptional analysis of murine [26,27], and human disc tissues [28]. This analysis identified 14 of 20 candidate genes, as well as numerous additional peptides belonging to related protein superfamilies. Specific examples include Wisp1, Dusp1 and NFAT5 identified in our proteomic study which are related to Wisp2, Dusp27 and NFATC1, respectively, which were previously identified at the level of gene expression in the developing mouse IVD [27]. This interesting trend may reflect temporal changes in the expression of related proteins associated with skeletal development and maturity, or the co-expression of multiple related proteins within the IVD and differences in the resolution or sensitivity of transcriptional and proteomic analysis. To further validate the list of proteins identified in the current study, we compared our findings to recent datasets generated from the proteomic analysis of human cartilaginous tissues, which included both NP and AF [18]. Similar to the analysis of human IVD tissues, we identified well-characterized ECM proteins (aggrecan, decorin, versican, lumican, type-I and type-II collagen) as well as intracellular proteins such as phospholipase A_2_ and protein S100-A9. However, some discrepancies were also noted. For example, high levels of lubricin expression were reported in human nucleus pulposus tissues; however, lubricin was not identified in the murine IVD. These discrepancies highlight the need for further investigation, specifically aimed at examining the proteomic signature of human and murine tissues at similar ages or stages of disc disease to more thoroughly assess conservation between species relevant to the use of mouse models to study human disc disease [35].

Our analysis also detected the expression of proteins previously found to be enriched in the murine developing node at embryonic day E8.5, including Cnot1, Myom1 and CA3. These findings validate studies by our lab and others that used lineage tracing to demonstrate that the murine nucleus pulposus of the IVD is derived from the embryonic notochord [36,37]. Our previous studies detected the presence of notochord cells within the mouse NP up to 9 months of age, therefore we anticipate that a population of notochord cells would be present in the NP at the time point examined in this study. The detection of notochord-specific markers in our analysis validates our ability to capture the multiple cell types that constitute the mature IVD. Further suggesting that our analysis included notochord or notochord-derived cells was the identification of proteins annotated to the protein-transport ATP synthase complex. Recent studies in the developing zebrafish demonstrated that vacuolated notochord cells require acidification, in the form of ATP/H^+^ complex to form and maintain vacuoles [38].

It is interesting to note that several proteins identified by our screen are associated with IVD-specific or skeletal phenotypes in mutant mice, such as Sparc [39,40] and Npr3 [41]. Sparc (secreted protein, acidic, and rich in cysteine) is a matricellular protein important for modulating interactions between cells and their ECM, including collagen deposition and remodeling, and growth factor efficacy. Targeted deletion of *Sparc* alters the cellular content and ECM composition/organization of the annulus fibrosus, and results in disc wedging, endplate calcification, and sclerosis [39], which lead to an increase in the detection of back pain-associated behaviour [40]. Similarly, mutation of natriuretic peptide receptor C (*Npr3*) resulted in thin or absent NP with calcification of the dorsal AF in mice at postnatal-day 21 [41]. Inactivation of two paralogous genes is often required to observe a developmental phenotype in mutant mice, as is the case with Sox5 and Sox6 [42,43]. Mice lacking expression of both *Sox5* and *Sox6* demonstrated defects in notochord sheath formation, leading to notochord cell apoptosis and the absence of NP within the IVD. We also detected IVD-enriched proteins such as FoxA2/Hnf-3β, which has been implicated in notochord development in other model organisms [44]. Other proteins detected in our study, such as Tgf-β, demonstrate general disruptions in skeletal development in knockout mouse models [45]. This further exemplifies that our proteomic characterization of the IVD provides insight into the homeostasis of the murine IVD and a list of candidate proteins that can be further explored to determine their role within this complex tissue.

Although accurate microdissection of distinct IVD tissue types is feasible in large animal models or human tissue samples, this technique is difficult in the mouse given the small size of the disc (2–4 mm in diameter) and the low yield of protein obtained from each tissue compartment. As such, the current study was designed to assess the intact IVD. Furthermore, since we aimed to examine cellular and extracellular proteins, experiments were designed with intact tissues as the starting material in lieu of isolated cell populations. This experimental approach also avoided potential misclassification of proteins to specific tissue types (*i*.*e*. NP *vs* AF *vs* CEP) that may result from the technical challenges associated with accurately isolating one tissue type from the other in the murine IVD. We acknowledge the limitations associated with the analysis of the IVD as a whole; however, by immunohistochemistry we have validated the tissue-specific patterns of protein expression thereby confirming that our list of identified proteins reflects all tissues types within the IVD (NP, AF and CEP).

## Conclusion

This study provides the first proteome database of IVD tissue from healthy, skeletally mature mice. The identification and classification of the proteins present within the healthy IVD as a whole is a critical first step in determining the pathways and processes that are required to maintain IVD homeostasis. Such data will establish a solid foundation for better understanding the complex microenvironment of the IVD, and provide a starting point from which to identify and ultimately target the pathways that are altered during the process of disc degeneration that contribute to back pain.

## Supporting Information

S1 Table Proteins from the murine intervertebral disc that were unidentified or classified as preliminary according to UniProt analysis. List of identified proteins from a skeletally mature CD-1 mouse IVD that were not annotated within the database at the time of analysis or that were considered putative uncharacterized (578), as well as any proteins that were derived from Ensembl automatic analysis pipeline and were therefore considered preliminary (396 proteins).
(PDF)

S2 Table Proteins identified in the murine intervertebral disc. List of identified proteins using the LC-ESI-MS/MS strategy from the IVD of skeletally mature (14 week old) wild-type CD-1 mice.
(PDF)
