# Supplementary material for: Proteomic Signature of the Murine Intervertebral Disc
Source: PLoS One. 2015 Feb 17;10(2):e0117807. doi: 10.1371/journal.pone.0117807 (PMC4331544; doi:10.1371/journal.pone.0117807)
Supplement: S1 Table — List of identified proteins from a skeletally mature CD-1 mouse IVD that were not annotated within the database at the time of analysis or that were considered putative uncharacterized (578), as well as any proteins that were derived from Ensembl automatic analysis pipeline and were therefore considered preliminary (396 proteins). (PDF) [file pone.0117807.s001.pdf]

Supplementary Table 1: Proteins identified that were considered preliminary according to Uniprot.

Uniprot Accession No.	Protein Name	% Coverage	No. Different Peptides
A2AAU3	Probable helicase with zinc finger domain	88.89	2
E9QM04	Fc receptor-like protein 6	77.78	2
D3Z184	Ropporin-1	68.85	3
E9Q7C4	Proline rich 14-like	68.85	3
A2AJH3	Glycylpeptide N-tetradecanoyltransferase 2	68.69	3
D3YTR0	Adenomatous polyposis coli protein 2	65.82	2
E9PZH6	WW domain-containing adapter protein with coiled-coil	65.82	2
E9PZ84	Tetratricopeptide repeat protein 16	60.86	2
D6Q0F7	Cytoplasmic dynein intermediate chain 2 isoform 2.5	57.01	2
D3YTN4	GTPase IMAP family member 4	53.62	4
E9PZ97	Bloom syndrome protein homolog	53.62	3
B1AQ57	Copper-transporting ATPase 2	48.82	2
D3YTR2	Receptor expression-enhancing protein 6	46.10	4
E9PZJ8	Activating signal cointegrator 1 complex subunit 3	46.10	2
E0CXJ6	Tubulin-specific chaperone E	45.00	2
E9QMU1	Kinesin-like protein KIF7	45.00	2
D3YUE4	Family with sequence similarity 151, member B	41.51	3
E2QRQ3	Double-stranded RNA-binding protein Staufen homolog 2	40.25	2
E9QNR8	4930432K21Rik	40.25	2
B1B1B8	Phosphatase and actin regulator 1	39.07	2
D3YUX1	Pogo transposable element with ZNF domain	38.26	4
E9Q1E1	Trophinin	38.26	4
D3YTT4	Isobutyryl-CoA dehydrogenase, mitochondrial	35.46	4
E9PZS7	Olfactory receptor 287	35.46	4
E9Q8P0	Troponin C, slow skeletal and cardiac muscles	35.04	2
D3YYQ4	Carbonic anhydrase 1	34.29	3
E9Q4S3	Ninein	34.29	3
E9PYJ9	LIM domain-binding protein 3	34.09	2
D3YUE7	GRAM domain-containing protein 4	33.67	3
E9PZX1	Gm17384	33.43	7
D3YTT9	ZZ-type zinc finger-containing protein 3	32.88	6
E9PXF0	Protocadherin 17	29.89	2
D3YTU3	Mina	29.68	5
E9PZW8	Unconventional myosin-IXb	29.68	5
D6RFP6	Dual-specificity testis-specific protein kinase 1	27.83	2
E9QLK3	BRCA1-associated ATM activator 1	27.83	2
E9PV45	Ubiquitin carboxyl-terminal hydrolase	27.27	2
E9Q2N7	Interferon-induced guanylate-binding protein 2	26.19	3
E9PW85	Inactive serine/threonine-protein kinase VRK3	24.82	2
D3Z7R1	Pyridoxal kinase	24.75	2
D3Z5P0	Serine/threonine-protein kinase BRSK1	24.42	2
E9QAF1	Spata31d1c	24.42	2
E9PUV9	Caspase recruitment domain-containing protein 10	24.32	2
D3YWN6	Protein downstream neighbor of Son	24.11	3

D3YYS9	Acid-sensing ion channel 4	23.81	2
E9Q4Z5	Gm6729	23.81	2
D3YX57	Fanconi anemia group I protein homolog	23.47	3
E9Q3G8	Nucleoporin 153	23.47	3
D3YWA6	Serine/threonine-protein kinase 33	22.69	2
E9Q2Z1	Cat eye syndrome chromosome region, candidate 2	22.69	2
B1AUY3	Rho GTPase activating protein 4	22.45	2
E9PYB0	AHNAK nucleoprotein 2	22.02	2
D3Z3A9	Reticulocalbin-3	20.28	3
E9Q8I5	Zinc finger protein 108	20.28	3
E0CY73	1700112E06Rik	20.21	2
E9QN41	Striatin-interacting proteins 2	20.21	2
D3YWY5	Mth938 domain-containing protein	18.71	4
E9Q3D6	Heat shock protein HSP 90-beta	18.71	4
D3YTR7	Adenylyl cyclase-associated protein	18.63	3
E9PZM4	Chromodomain helicase DNA binding protein 2	18.63	2
D3Z5N5	1-phosphatidylinositol 3-phosphate 5-kinase	18.49	3
E9QAE4	Nsd1	18.49	3
E9QKK1	Centromere-associated protein E	17.74	2
E9Q072	Selection and upkeep of intraepithelial T-cells protein 5	17.67	9
D3Z678	Gm15448	17.50	2
E9Q7D5	Rho guanine nucleotide exchange factor (GEF) 5	17.23	2
D3YUF9	UDP-glucuronic acid decarboxylase 1	16.76	3
D3Z0D4	DNA-binding protein Rfx5	16.67	2
E9Q6C7	Latrophilin-2	16.67	2
E0CYZ9	E3 ubiquitin-protein ligase RNF6	15.93	2
D3YYQ8	Dynein, axonemal, heavy chain 10	15.44	2
E9PXP7	Stonin-2	14.88	2
D3Z2Q3	Diablo homolog, mitochondrial	14.22	2
E9Q8A3	Phosphatidylinositol 4-kinase beta	14.22	2
D3Z7J9	60 kDa heat shock protein, mitochondrial	14.11	2
E9PXX8	Macc1	13.89	2
D3YX99	Subunit 5, mitochondrial	13.51	5
D6REH1	Peroxisomal membrane protein 2	13.44	2
E9QLA5	Inverted formin-2	13.44	2
B1AUY7	N-alpha-acetyltransferase 10	13.39	2
D3Z7F0	L-lactate dehydrogenase	13.32	2
A2AF47	Dedicator of cytokinesis protein 11	13.22	2
D3YYD9	Gfpt1	13.16	3
E9Q4F8	Ankyrin repeat domain 11	13.16	3
D3Z1N2	RING finger protein 10	13.15	2
E9Q7R9	WD repeat domain 96	13.15	2
D3Z7D5	Collagen alpha-2(VI) chain	12.64	2
D3YYI9	Gm7347	12.54	3
E9Q4H1	Adenomatous polyposis coli protein	12.54	3
E0CYH7	Filamin A-interacting protein 1-like	12.17	2
D3YYK2	Beta-defensin 30	11.80	3
D3Z2G7	Histone deacetylase 11	11.76	2

E9Q813	Ras-GEF domain-containing family member 1B	11.76	2
D3YVJ8	Retinol dehydrogenase 13	11.70	2
D3Z460	Gm1330	11.66	2
D3YU67	Vacuolar protein sorting-associated protein 8 homolog	11.61	3
E9Q0W7	cAMP-specific 3',5'-cyclic phosphodiesterase 7B	11.61	3
E9PWQ6	Serine/threonine-protein kinase 31	11.58	2
E0CY56	SUMO-interacting motifs containing 1	11.54	2
E9Q7G0	Nuclear mitotic apparatus protein 1	11.38	3
Q003Y8	Uncharacterized protein KIAA2022	11.37	4
D3YYY8	Rho guanine nucleotide exchange factor (GEF) 26	11.13	2
E9Q512	Thyroid hormone receptor interactor 11	11.13	4
B1AV28	Gm14862	11.07	2
D3Z2E7	AI607873	11.07	3
E0CXG4	Greb1	10.72	2
D6RH44	NMDA receptor-regulated protein 2	10.61	2
D3YZ06	Heat shock protein beta-1	10.59	3
E0CYV9	Protein 1110002E22Rik	10.09	2
D3YX91	Gm7682	10.02	2
D3Z061	Ubiquitin-like modifier-activating enzyme 6	9.93	3
E9Q640	RNA-binding protein 26	9.93	3
E9QM06	Telomeric repeat-binding factor 2	9.91	2
A2AQA7	Intron-binding protein aquarius	9.88	2
D6RH34	Phosphatidate phosphatase LPIN2	9.82	2
E9PX70	Collagen alpha-1(XII) chain	9.62	2
D3Z7F6	Cytochrome P450, family 2, subfamily t, polypeptide 4	9.57	2
E0CY67	Sodium/hydrogen exchanger 9B1	9.49	2
E9QN32	LIM/homeobox protein Lhx6	9.49	2
D3Z4T1	CUGBP, Elav-like family member 5	9.45	2
E9Q9S8	Glutamine repeat protein 1	9.45	2
E9Q1N6	RasGEF domain family, member 1A	9.41	2
D3Z0Q6	Glycogen [starch] synthase, muscle	9.40	2
E9Q7C3	Plekhg4	9.39	2
D3Z7I5	RAD51-associated protein 1	9.38	2
E9QKX5	Vacuolar protein sorting-associated protein 13B	9.38	2
E9QMF5	Neuron navigator 3	9.31	2
D3YVP6	Glutathione S-transferase Mu 7	9.27	2
E9Q264	Myosin, heavy chain 15	9.27	2
D3Z6W2	Tyrosine-protein phosphatase non-receptor type	9.25	2
E9PV87	TALPID3	9.24	2
E9PVY8	Microtubule-actin cross-linking factor 1	9.18	2
E9PWK2	Vomeronasal 1 receptor 224	9.16	2
D3YUE0	Three prime repair exonuclease 1	9.10	2
A2AJG1	Acyl-CoA-binding domain-containing protein 7	9.08	2
D3YXJ0	Diacylglycerol kinase, eta	9.05	4
E9PZ36	Polycystic kidney and hepatic disease 1	8.90	2
D6RH66	Transmembrane and TPR repeat-containing protein 4	8.82	2
E9Q7K7	Janus kinase and microtubule-interacting protein 3	8.65	3
E9QLT6	Aryl hydrocarbon receptor nuclear translocator	8.60	2

D3Z757	WD repeat domain 86	8.50	2
D3Z2A3	Histone demethylase UTY	8.47	2
D3Z6Z5	Family with sequence similarity 189, member A1	8.47	3
E9Q7Y3	Uncharacterized protein ENSP00000471857 homolog	8.47	2
E9QKE4	Rab3 GTPase-activating protein non-catalytic subunit	8.47	3
E9Q9F1	Kcnq5	8.45	2
D6RFA3	Hexokinase-3	8.42	2
E0CYB9	Mitochondrial fission factor	8.38	2
E9QN53	Lebercilin-like protein	8.38	2
D6RCW6	V-type proton ATPase subunit C 2	8.34	2
A2AHD2	Protein Kcnk15	8.32	2
D6RGU3	Dedicator of cytokinesis protein 2	8.29	2
E9QLW6	Microtubule-associated serine/threonine-protein kinase 2	8.29	2
D6RH37	Serine/threonine-protein kinase N1	8.25	2
D3Z795	Proteasome assembly chaperone 1	8.13	2
E9PWI6	GRIP and coiled-coil domain-containing protein 2	8.13	2
E9QKS6	Synaptotagmin-like protein 2	8.13	2
D6RG93	Probable tubulin polyglutamylase TTLL9	8.08	2
A2AF67	Dedicator of cytokinesis protein 11	7.82	2
E9Q357	Gm17673	7.82	3
D3YVM5	60S acidic ribosomal protein P0	7.77	3
D3Z3N9	Nyap2	7.77	3
E9Q8N1	Titin	7.77	3
E9PY76	Gm4868	7.53	2
D3Z2W1	Diacylglycerol kinase, iota	7.27	2
E9Q8E3	ABI gene family, member 3 (NESH) binding protein	7.27	2
D3YWG0	11-cis retinol dehydrogenase	7.21	2
E9Q309	Centrosomal protein 350	7.21	2
D3Z5Q9	Tropomodulin-4	7.16	2
D3Z3F0	Lateral-signaling target protein 2 homolog	7.12	2
A2AM74	Kinesin-like protein KIF17	7.09	2
D3Z2B6	Vomeronasal 2, receptor 35	7.02	2
E9Q804	Ankyrin repeat domain-containing protein 17	7.02	2
E9PUU8	Fibrocystin-L	7.00	2
D6RFS3	Chloride channel protein 2	6.99	2
E9PYI8	Ubiquitin specific peptidase 14	6.91	2
D3Z4D4	1700064H15Rik	6.88	2
E0CY47	Plch1	6.75	2
D3YVU7	Lysine-specific demethylase 2B	6.74	4
E9Q2H1	E3 ubiquitin-protein ligase UBR5	6.74	4
B1AVN8	Phosphatase and actin regulator 2	6.60	2
D3Z440	COP9 signalosome complex subunit 7a	6.60	2
D6RGL4	Ankyrin repeat domain-containing protein 16	6.60	2
E9Q8Y0	Myl2	6.60	2
D3Z709	Disks large-associated protein 1	6.47	2
D3Z1M7	Synaptojanin-1	6.33	2
E9Q7Q0	Mucin-4	6.33	2
A2AP83	Fibronectin type III domain containing 3C2	6.32	2

E9QAU4	Sickle tail protein	6.28	3
A2ANY6	Midasin	6.24	2
D3Z0B2	Nuclear-interacting partner of ALK	6.20	2
E9Q6C1	Oxysterol-binding protein	6.20	2
D3Z0I9	Rnft2	6.16	4
E9Q6J4	Ceacam3	6.16	4
D3YXG0	Hemicentin 1	6.11	2
E9PVU7	cAMP-specific 3',5'-cyclic phosphodiesterase 4D	6.08	2
E9QQ17	Lipid phosphate phosphatase-related protein type 2	6.08	2
E0CZ72	Kinesin-like protein KIF2A	6.06	2
B1AUX2	Host cell factor 1	5.89	2
D3Z784	Probable UDP-sugar transporter protein SLC35A5	5.84	2
E9QKK8	Probable phospholipid-transporting ATPase 11C	5.84	2
A2AGR6	DNA damage-binding protein 2	5.83	2
D6RGN2	Plcd4	5.76	2
D3YXQ5	Probable phospholipid-transporting ATPase ID	5.75	2
D3Z6W1	Microtubule-associated protein 6	5.75	2
E9Q423	Dysferlin	5.75	2
E9QKD1	Nucleolar protein 8	5.75	2
D3YV10	Coiled-coil domain containing 13	5.61	2
E9Q1N0	4932431P20Rik	5.61	2
D3Z7A8	WD40 repeat domain 95	5.57	2
D3YXM6	Yip1 domain family, member 3	5.53	2
E9Q3Z5	Supervillin	5.53	2
D6RFN6	Caspase 2, apoptosis-related cysteine peptidase	5.46	2
E9QLJ0	Cardiomyopathy-associated protein 5	5.46	2
D3Z0R4	Tubby-related protein 2	5.35	3
D3Z576	Filamin-C	5.27	2
E9QA45	GTP-binding protein 2	5.27	2
E9Q4N3	E3 ubiquitin-protein ligase LNX	5.21	3
E9QPZ3	Filaggrin-2	5.19	2
D3YZ35	Neurofilament medium polypeptide	5.17	2
D3Z3M7	CAP-Gly domain-containing linker protein 1	5.03	3
E9Q8M8	Mineralocorticoid receptor	5.03	3
D3Z041	Long-chain-fatty-acid--CoA ligase 1	5.01	3
E0CYG5	Transmembrane protein 191C	4.99	2
E9QN87	Plectin	4.99	2
E9PXR3	Sestrin-1	4.97	2
E9Q8S5	Microtubule-associated serine/threonine-protein kinase 3	4.96	3
D3Z7A7	Formin-like protein 3	4.93	2
E9PZ38	Serine/threonine-protein kinase WNK4	4.93	2
E0CYY1	Anamorsin	4.86	2
D3Z0Z4	ATP-binding cassette sub-family G member 3	4.84	2
E9Q793	4931429I11Rik	4.84	2
D3YWI1	Fructose-bisphosphate aldolase	4.77	2
D3Z736	L-lactate dehydrogenase	4.74	2
A2AHW8	GTPase-activating Rap/Ran-GAP domain-like protein 3	4.71	2
D3Z4K0	Ankyrin repeat domain 36	4.70	2

E9PX39	Adamts14	4.67	2
D3Z7F5	NACHT, LRR and PYD domains-containing protein 9A	4.65	3
E9QKV8	Nrde-2	4.65	3
E9QMD3	Zinc finger homeobox protein 3	4.65	2
E9PYK6	Olfactory receptor 1410	4.62	2
E9PZ41	Trafficking protein particle complex subunit 9	4.59	2
E9Q8N4	Microtubule-associated tumor suppressor 1 homolog	4.52	2
E0CXW4	Histone-lysine N-methyltransferase SUV420H2	4.50	2
E0CZF7	Homeobox protein prophet of Pit-1	4.49	2
D3YU01	Plekha1	4.46	4
D3YXX8	TP53RK-binding protein	4.40	2
D3YZW1	SLIT-ROBO Rho GTPase-activating protein 1	4.40	5
E9Q448	Tropomyosin alpha-1 chain	4.40	2
A8DUV1	Hemoglobin alpha, adult chain 1	4.38	2
E9PWB8	Tubulin polyglutamylase TTLL7	4.38	2
D3YXL0	Coiled-coil domain containing 170	4.35	2
E9Q3Y8	Gm6811	4.35	2
D3YUC3	1700026D08Rik	4.32	8
E9QNY8	Sacsin	4.31	2
E9PWG4	Myosin light chain 1/3, skeletal muscle isoform	4.27	2
D6RG56	Round spermatid basic protein 1	4.26	2
D6RIK9	Rpgrip1-like	4.24	2
A2AEP5	ATP-binding cassette sub-family A member 5	4.23	2
D3YVU6	Partner and localizer of BRCA2	4.20	2
D3YX59	Nicotinamide N-methyltransferase	4.18	2
E9Q3H7	Dynein, axonemal, heavy chain 6	4.18	2
D3YXI6	N-lysine methyltransferase SETD8	4.15	4
D3YVM9	Setd8	4.12	2
E0CY48	Tetratricopeptide repeat protein 39B	4.12	2
E9Q1W3	Nebulin	4.12	2
D3YTW9	Wnt5b	4.10	2
E9Q455	Tropomyosin alpha-1 chain	4.10	2
D3YXH8	Integrin beta 2-like	4.09	2
E9Q3U6	Killer cell lectin-like receptor subfamily B member 1C	4.09	2
E0CXJ0	Slco6d1	4.03	2
D3YZH8	Transducin beta-like protein 2	4.00	2
E0CXX5	Coiled-coil domain containing 19	4.00	2
E9Q5D9	Insulin-like growth factor-binding protein 7	4.00	2
D3Z5G8	Disco-interacting protein 2 homolog B	3.98	3
E9QAD4	Coiled-coil domain-containing protein 93	3.98	3
E9QPR6	Transmembrane protease serine 13	3.96	2
A2ANT9	Ankyrin repeat and SAM domain-containing protein 6	3.90	2
D6REH6	BCL-6 corepressor-like protein 1	3.90	2
E9PU96	Nucleolar pre-ribosomal-associated protein 1	3.90	2
E9QP09	Cullin-9	3.90	2
E9PX79	March10	3.88	2
D3YUU6	Cornulin	3.75	2
D3Z5G3	Anion exchange protein 2	3.75	3

E9QNW4	Piezo-type mechanosensitive ion channel component 2	3.72	2
A2AHQ2	G-protein-coupled receptor 64	3.58	2
D3Z5T8	4930407I10Rik	3.57	2
E9QAX1	Balap2l2	3.57	2
D3Z0L7	SUN domain-containing protein 1	3.55	2
E9Q6J5	Biorientation of chromosomes in cell division 1-like	3.55	2
D3Z0G8	MAGUK p55 subfamily member 4	3.47	2
E9Q6D8	Complement component 6	3.47	2
D3Z4T0	Interleukin-17 receptor B	3.46	3
E9PYT0	Rho GTPase-activating protein 5	3.36	2
D3Z4U7	Testis-expressed sequence 35 protein	3.35	3
E0CZ30	Myosin light chain 1/3, skeletal muscle isoform	3.30	2
E9PW83	Fam184a:family with sequence similarity 184, member A	3.30	2
E9QNG1	Intersectin-2	3.30	2
D3YZR0	Gm15737	3.28	2
D3Z4D9	C2 domain-containing protein 3	3.28	2
E9Q9B2	NEDD4 binding protein 2	3.28	2
D3YUZ4	CREB/ATF bZIP transcription factor	3.27	2
D6REG6	PX domain-containing protein 1	3.26	2
E9QPI5	Sister chromatid cohesion protein PDS5 homolog A	3.26	2
D3Z5W6	Fatty acyl-CoA reductase 1	3.21	2
E9QAZ9	Vomeronasal 2, receptor 100	3.21	2
A2AG09	RNA-binding protein 41	3.17	2
E9Q6T9	BTB/POZ domain-containing protein KCTD1	3.16	2
D3YYR3	2210418O10Rik	3.11	2
E9PUJ6	Probable E3 ubiquitin-protein ligase MYCBP2	3.10	2
E9QPG2	Polycystic kidney disease protein 1-like 2	3.10	2
E9QL22	TatD DNase domain containing 3	3.04	2
E9PVU0	Unconventional myosin-VI	3.03	2
E9PXA7	Cytosolic carboxypeptidase 1	3.01	2
D3YY55	Histone deacetylase complex subunit SAP18	2.98	3
E9Q456	Tropomyosin alpha-1 chain	2.98	3
E9PYP7	Trehalase	2.86	2
D3YYC3	Histone-lysine N-methyltransferase SETDB1	2.85	3
E9PUA3	IQ motif and SEC7 domain-containing protein 1	2.83	2
E9QP46	Nesprin-2	2.83	2
A2ALV5	Protein AI481877	2.76	2
E9PYD7	Kelch repeat and BTB (POZ) domain containing 6	2.76	2
D3YXT2	Ribosomal protein L10A, pseudogene 2	2.72	2
E9Q444	Zinc finger and BTB domain containing 21	2.72	2
E0CZA1	T-complex protein 1 subunit epsilon	2.68	2
D3Z4N8	Vomeronasal 2, receptor 72	2.66	2
E9Q9M0	Centrosomal protein of 290 kDa	2.66	2
E9PW44	YTH domain containing 1	2.60	2
E9PUV7	Gm872	2.56	2
E9Q616	AHNAK nucleoprotein (desmoyokin)	2.56	4
D3Z3X6	Latrophilin-3	2.49	4
D3YYD6	Aquaporin-9	2.47	2

E9Q489	Calsequestrin	2.47	2
D3YZZ5	Tmed7	2.40	2
E9Q5L3	Acadsb	2.40	2
D3YZE8	Cdca2	2.35	3
E9PUL4	Pde8b	2.35	2
E9Q559	Sarcoplasmic/endoplasmic reticulum calcium ATPase 3	2.35	3
D3YY08	Tubulin polyglutamylase TTLL4	2.32	2
E9Q452	Tropomyosin alpha-1 chain	2.32	2
E9PWG6	Non-SMC condensin I complex, subunit G	2.30	2
E9QP99	Golgin subfamily A member 3	2.26	2
E9Q9D8	ankyrin repeat domain 35	2.24	3
D3Z2J3	Putative ATP-dependent RNA helicase DHX30	2.23	2
E9Q7S1	Zinc finger protein 106	2.23	3
D3Z3B2	V-type proton ATPase 16 kDa proteolipid subunit	2.22	2
D6RIM8	Harmonin	2.16	2
A2AE98	WD and tetratricopeptide repeats protein 1	2.12	2
E9Q7E2	AT rich interactive domain 2 (ARID, RFX-like)	2.11	3
D3YWV3	Differentially-expressed in FDCP 8	2.09	4
E9Q390	Myoferlin	2.09	7
D3Z2L1	General transcription factor 3C polypeptide 2	2.08	2
E9Q892	Lrch3	2.08	2
D3Z6N0	BB014433	2.06	3
E9Q1K1	4933411K20Rik	1.87	2
B1ASL6	Tumor necrosis factor receptor superfamily member 18	1.85	2
E9PZ74	Vomeronasal 2, receptor 7	1.85	2
D3Z030	Leucine-rich repeat-containing protein 16A	1.82	2
D3Z007	Interleukin-1 receptor-associated kinase 3	1.81	3
E9Q5M6	WD repeat domain 52	1.81	3
E9QQ10	A-kinase anchor protein 9	1.80	2
E9QML5	Zinc finger protein 638	1.76	2
E9Q0D4	SET binding factor 2	1.75	2
D3Z5A7	Peroxisome biogenesis factor 1	1.62	3
E9QAC0	StAR-related lipid transfer protein 9	1.62	3
D3Z0T2	Dimethylaniline monooxygenase [N-oxide-forming] 1	1.60	2
E9Q6Y8	Ubiquitin specific peptidase 31	1.60	2
D3Z0I5	Tripartite motif-containing protein 59	1.53	2
D3Z5R8	40S ribosomal protein S19	1.48	3
E9QAH1	Golgi autoantigen, golgin subfamily b, macrogolgin 1	1.48	3
E0CZ42	Fer-1-like 6 (C. elegans)	1.44	2
E9PUJ2	DNA repair protein RAD50	1.44	2
D3Z1X5	Rho GTPase-activating protein 26	1.42	2
E9Q7S8	Vomernonasal 2, receptor 22	1.42	2
D3YU32	Gm362	1.40	3
D3Z533	Galntl5	1.40	3
E9Q0A4	Kinesin-like protein KIF21B	1.40	3
E9QA22	Xinc finger protein 644	1.40	3
D3Z2S4	Enolase	1.38	3
E9Q8C5	Family with sequence similarity 3, member C	1.38	3

A2AEC2	Transcription elongation factor A protein-like 3	1.24	2
D3Z4B5	Family with sequence similarity 210, member A	1.24	3
E9QKH0	CLIP-associating protein 1	1.23	2
D6RFE3	Transmembrane protein 180	1.19	2
E9Q555	E3 ubiquitin-protein ligase RNF213	1.17	5
D6RFR7	DCC-interacting protein 13-beta	0.98	2
E9Q7N9	Dynein, axonemal, heavy chain 11	0.98	2
E9QLK7	Transformation/transcription domain-associated protein	0.98	2
D3Z6P9	DEAD (Asp-Glu-Ala-Asp) box polypeptide 43	0.91	2
E9QK83	Small subunit processome component 20 homolog	0.91	2
D3Z4A4	Peroxiredoxin-2	0.88	2
D3YV81	Sarcolemmal membrane-associated protein	0.86	2
D3Z667	Dynein heavy chain 2, axonemal	0.86	2
D3Z6P1	Multifunctional protein ADE2	0.74	2
E9QK82	Myelin protein P0	0.74	2
D3YVW2	Golgi integral membrane protein 4	0.30	5
