# Supplementary material for: Proteomic Signature of the Murine Intervertebral Disc
Source: PLoS One. 2015 Feb 17;10(2):e0117807. doi: 10.1371/journal.pone.0117807 (PMC4331544; doi:10.1371/journal.pone.0117807)
Supplement: S2 Table — List of identified proteins using the LC-ESI-MS/MS strategy from the IVD of skeletally mature (14 week old) wild-type CD-1 mice. (PDF) [file pone.0117807.s002.pdf]

Supplementary Table 2 - Identified proteins within the intervertebral disc.

Uniprot Accession No.	Protein Name	% Coverage	No. Different Peptides
B2RXU7	Chondroitin sulfate synthase 3	97.87	2
B9EI21	Zinc finger matrin type 3	82.61	2
B9EKB8	Insulin-like growth factor 2 receptor	65.82	4
P46660	Alpha-internexin	65.82	2
Q9JIH4	Nuclear factor of activated T cells 5	63.37	2
**Q8BSS6**	**Transcription factor SOX-6**	**57.69**	**3**
Q91VH3	Tropomyosin 2, beta	57.36	3
D6Q0F7	Cytoplasmic dynein intermediate chain 2 isoform 2.5	57.01	2
Q64343	ATP-binding cassette sub-family G member 1	57.01	2
Q8C0Q3	Tetratricopeptide repeat domain 34	50.81	2
B1AXJ0	5-azacytidine-induced protein 1	50.00	2
Q505F0	Asap1	49.28	2
P59528	Taste receptor type 2 member 123	49.28	2
P61164	Alpha-centractin	49.06	2
Q6P8J7	Creatine kinase S-type, mitochondrial	46.94	2
A4Q9E4	Probable tubulin polyglutamylase TTLL2	46.43	2
E9PZJ8	Activating signal cointegrator 1 complex subunit 3	46.10	2
Q8BYN3	Inositol-tetrakisphosphate 1-kinase	45.95	2
P31725	Protein S100-A9	44.03	13
P63101	14-3-3 protein zeta/delta	41.51	3
B2RRF6	Zinc finger protein 518A	40.94	2
B7ZND3	Avian erythroblastosis virus E-26 (v-ets) oncogene related	40.41	4
Q7TQ48	Sarcalumenin	40.11	4
P40142	Transketolase	39.73	12
Q5U440	Maternal embryonic leucine zipper kinase	39.72	2
Q8K0Z9	Probable G-protein coupled receptor 153	38.60	2
Q6PAJ3	GRB2-associated and regulator of MAPK protein-like	38.46	2
Q0VBN2	Dermatan-sulfate epimerase-like protein	38.16	2
Q9DCC3	Coiled-coil domain-containing protein 107	37.50	2
B2RSN6	REST corepressor 3	37.41	2
A6ZI46	Fructose-bisphosphate aldolase	37.06	2
E9QMK7	Arap3	37.06	2
D9HP81	Regulating synaptic membrane exocytosis 2	37.06	2
Q9D6J4	N-terminal EF-hand calcium-binding protein 3	36.42	2
P10639	Thioredoxin	34.09	2
A2RSS2	Carbohydrate sulfotransferase 10	33.95	2
E9Q165	Trpm8	33.67	3
Q02242	Programmed cell death protein 1	33.17	3
Q8CGY6	Protein unc-45 homolog B	31.71	3
Q9WV42	Nuclear receptor coactivator 4	31.06	2
P60879	Synaptosomal-associated protein 25	30.57	2
Q9D5R3	Centrosomal protein of 83 kDa	30.09	2
P07759	Serine protease inhibitor A3K	29.89	2
A2A864	Integrin Beta 4	29.26	2

Q14B66	Potassium voltage-gated channel, subfamily Q, member 3	29.25	2
Q9DAM4	Citrate synthase like	29.25	2
P97864	Caspase-7	29.17	2
Q5F4S6	Trpm3	29.02	2
Q8K2Y2	Receptor-interacting serine-threonine kinase 3	28.87	3
Q9WVM2	Dematin	28.57	2
Q8R4D4	Signal transducer and activator of transcription 6	27.22	3
D3YU07	RAD50 interactor 1, isoform CRA f	26.60	6
**D3YW52**	**Alpha-2-macroglobulin**	**26.19**	**3**
Q5FWH3	Grainyhead-like protein 3 homolog	26.10	5
A6H694	Leucine-rich repeat-containing protein 63	26.09	2
O70343	PPAR-gamma coactivator 1-alpha	26.09	2
A2A9Z0	Dystrophin	25.93	2
Q0P661	Proline rich Gla (G-carboxyglutamic acid) 1	25.38	2
C6EQG1	Ubiquitin carboxyl-terminal hydrolase 29	25.12	2
O54949	Serine/threonine-protein kinase NLK	24.82	2
E9QL17	Serine/threonine-protein kinase MARK1	24.75	2
Q8BH44	Coronin-2B	24.71	2
Q9WV02	RNA-binding motif protein, X chromosome	24.67	2
Q4FK59	Enolase 3, beta muscle	24.66	3
O55226	Chondroadherin	24.24	2
Q9DCM0	Persulfide dioxygenase ETHE1, mitochondrial	24.22	2
P08249	Malate dehydrogenase, mitochondrial	24.16	2
A2A791	Zinc finger MYM_type protein 4	24.11	2
Q3KNG4	Neuropeptide FF-amide peptide precursor	24.09	2
B2RWR1	Protocadherin 20	23.97	2
Q2KHI9	DNA helicase MCM9	23.28	2
Q00898	Alpha-1-antitrypsin 1-5	23.08	2
Q9D5U0	Lysophosphatidylcholine acyltransferase 2B	22.90	2
Q3UGP9	Leucine-rich repeat-containing protein 58	22.89	2
B2RY08	Dedicator of cytokinesis 9	22.64	2
P80316	T-complex protein 1 subunit epsilon	22.61	2
P80316	T-complex protein 1 subunit epsilon	22.61	2
Q8VDD5	Myosin-9	22.45	2
Q45VK6	Interleukin enhancer binding factor 3	22.06	2
P70270	DNA repair and recombination protein RAD54-like	22.04	2
Q80XH2	Interphotoreceptor matrix proteoglycan 2	22.03	2
P09411	Phosphoglycerate kinase 1	22.02	2
A7TU71	Shroom2	21.60	2
A2ARV4	Low-density lipoprotein receptor-related protein 2	21.54	2
Q80U35	Rho guanine nucleotide exchange factor (GEF) 17	21.16	4
Q9ESP5	Fez family zinc finger protein 2	20.89	2
**Q04887**	**Transcription factor SOX-9**	**20.75**	**3**
Q80ZE2	Sialic acid-binding Ig-like lectin 5	20.63	2
P99027	60S acidic ribosomal protein P2	20.38	2
A0JNY8	RE1-Silencing Transcription Factor	20.20	2
Q7TPD5	Ribosomal protein S6 kinase	20.19	2
B1AVI6	Sushi-repeat-containing protein, X-linked 2	20.00	3
Q2EY15	Protogenin	19.88	2

Q5SDA5	Retinal guanylyl cyclase 2	19.76	2
B7ZMQ1	CD84	19.52	2
A2ABQ3	Centrosomal protein of 112 kDa	19.46	2
Q8CCJ3	E3 UFM1-protein ligase 1	19.35	2
Q5VCS6	Tudor domain-containing protein 5	19.19	2
Q3TXI6	Secreted acidic cysteine rich glycoprotein	19.14	2
Q99MX7	Cat eye syndrome critical region protein 6 homolog	18.53	2
B7ZN98	Otoferlin	18.49	2
B2RPU9	Neuromedin U receptor 2	18.47	2
Q4FJU1	Serine (or cysteine) peptidase inhibitor, clade E, member 2	18.44	3
Q8CIA8	Uaca	18.18	2
Q61785	ORF2	17.99	2
D3Z750	Maestro heat-like repeat-containing protein family member 2A	17.74	2
E9QJR6	Polycystin-1	17.73	2
D3YU22	LIM and calponin homology domains-containing protein 1	17.67	2
D3YU22	LIM and calponin homology domains-containing protein 1	17.67	2
E9QK00	Tight junction protein ZO-1	17.50	2
Q9EP71	Ankycorbin	17.35	2
Q6DR98	Neuregulin-1 type I beta1-a	17.24	5
B2RQL1	Coiled-coil domain containing 158	17.22	2
Q9Z285	Tektin 1	17.21	2
Q3U186	Probable arginine--tRNA ligase, mitochondrial	17.15	2
Q99K08	6-phosphofructokinase	17.13	3
Q3U1F0	Ligase III, DNA, ATP-dependent	17.09	2
Q9D738	Ankyrin repeat and SOCS box protein 12	16.93	2
B2RX00	RB1-inducible coiled-coil 1	16.87	2
Q9D5T7	HORMA domain-containing protein 1	16.81	2
Q6NZC3	Solute carrier family 30 (zinc transporter), member 3	16.67	2
Q7TN79	A-kinase anchor protein 7 isoform gamma	16.64	3
Q9QWT9	Kinesin-like protein KIFC1	16.62	2
Q6NZR2	Myb/SANT-like DNA-binding domain-containing protein 2	16.55	2
B2RWR3	Coiled-coil domain containing 73	16.38	2
Q5FW75	Actinin alpha 2	16.31	2
Q5PR68	Centrosomal protein of 112 kDa	16.19	2
Q9WU03	Kunitz-type protease inhibitor 2	16.19	2
Q7TSV6	Putative aspartate aminotransferase, cytoplasmic 2	16.18	3
Q7TRL9	Olfactory receptor 745	15.79	2
B9EKQ3	PR domain containing 2, with ZNF domain	15.63	2
A2A7Z8	Arylacetamide deacetylase-like 3	15.61	2
Q71LX8	Heat shock protein 84b	15.54	2
P56480	ATP synthase subunit beta, mitochondrial	15.28	2
P58873	Rhomboid-related protein 3	15.28	2
Q3KPE4	Phospholipase C, zeta 1	15.20	2
A0JP62	F-box and WD-40 domain protein 13	15.14	2
B2BBR8	Xin actin-binding repeat containing 2	15.10	2
Q148R9	Regulator of G-protein signaling 9-binding protein	15.04	2
Q7TPV4	Myb-binding protein 1A	15.02	2
Q9EQ38	Vomeronasal receptor V1RC5	15.01	2
Q8CEH8	Profilin	14.96	2

Q9CQL5	39S ribosomal protein L18, mitochondrial	14.94	2
Q9D3P1	Trichohyalin-like protein 1	14.94	2
Q3TGF2	Family with sequence similarity 107, member B	14.93	2
Q14DL3	Leucine-rich repeat and IQ domain-containing protein 3	14.90	3
P08553	Neurofilament medium polypeptide	14.88	2
Q14AV0	Myosin binding protein H	14.84	2
Q91YT9	6-phosphogluconate dehydrogenase, decarboxylating	14.76	3
Q9D9J3	Actin-related protein T1	14.73	2
Q9CZA6	Nuclear distribution protein nudE homolog 1	14.71	2
Q9DBA6	Peroxisomal leader peptide-processing protease	14.64	2
B2RWW8	Myosin, heavy polypeptide 8, skeletal muscle, perinatal	14.64	2
Q80VZ1	Calpain 6	14.49	2
Q3UHI0	Serine-rich coiled-coil domain-containing protein 2	14.22	2
Q8R554	OTU domain-containing protein 7A	14.20	2
A2AHD1	WNT1 inducible signaling pathway protein 2, isoform CRA_a	14.11	2
Q640N8	Major intrinsic protein of eye lens fiber	14.00	2
A1X3U7	Beta growth arrest specific protein 11	13.98	2
P09036	Serine protease inhibitor Kazal-type 3	13.89	2
Q9Z0U9	Sphingosine 1-phosphate receptor 3	13.87	2
A2AQP0	Myosin-7B	13.75	2
A8C756	Thyroid adenoma-associated protein homolog	13.72	2
A2A6J1	Troponin T, fast skeletal muscle	13.65	2
Q9CYU9	PRP18 pre-mRNA processing factor 18 homolog (yeast)	13.64	2
B2RU94	Olfactory receptor 1115	13.63	2
Q80UL7	Heart and neural crest derivatives expressed transcript 1	13.60	4
Q8BI29	Specifically androgen-regulated gene protein	13.56	4
Q6T707	Stearoyl-coenzyme A desaturase 4	13.55	6
Q8BJF9	Charged multivesicular body protein 2b	13.53	3
Q14AY4	Mitogen-activated protein kinase kinase kinase 5	13.50	2
Q8R5B7	General transcription factor IIF subunit 1	13.47	3
Q8R480	Nuclear pore complex protein Nup85	13.45	2
Q78KN5	Leucine rich repeat containing 40	13.38	2
A2AFF9	Plexin-B3	13.35	3
Q80UF7	TIR domain-containing adapter molecule 1	13.25	4
Q8BW94	Dynein heavy chain 3, axonemal	13.23	2
A2AF47	Dedicator of cytokinesis protein 11	13.22	2
Q99KR8	Plasma alpha-L-fucosidase	13.16	2
B2RU58	G-protein-coupled receptor 115	13.11	2
Q6XBG3	ATP-binding cassette transporter sub-family A member 14	13.05	2
Q9JI67	Beta-1,3-galactosyltransferase 5	12.92	2
Q9D881	Cytochrome c oxidase subunit 5B, mitochondrial	12.90	2
Q9CRY7	Gdpd1	12.88	2
Q3TE63	Peptidyl-prolyl cis-trans isomerase A	12.85	2
Q8BKU8	Transcription factor HES-7	12.84	4
Q9QXE7	F-box-like/WD repeat-containing protein TBL1X	12.77	2
B1AU57	Envoplakin	12.73	2
Q8BG58	Transmembrane prolyl 4-hydroxylase	12.72	5
Q6PGB9	A kinase (PRKA) anchor protein 6	12.68	2
E9QKU0	Intraflagellar transport protein 88 homolog	12.64	2

P42859	Huntingtin	12.59	2
Q9Z218	Dipeptidyl aminopeptidase-like protein 6	12.55	2
Q923C5	Centromere protein B	12.53	2
Q8VFI0	Olfactory receptor 788	12.52	3
A2AAA9	C-type mannose receptor 2	12.50	2
D7RXM0	GPR155 variant 4	12.50	2
A3KGK3	Fer-1-like protein 4	12.41	2
Q9QZS8	SH2 domain-containing protein 3C	12.39	2
Q8K2W0	Collagen, type IX, alpha 2	12.38	3
Q9DCU6	39S ribosomal protein L4, mitochondrial	12.28	2
P97457	Myosin regulatory light chain 2, skeletal muscle isoform	12.23	3
Q6PER8	Myosin VB	12.22	2
Q149C8	Hexokinase 3	12.15	2
P60882	Multiple epidermal growth factor-like domains protein 8	12.09	3
Q45VK7	Dynein cytoplasmic 2 heavy chain 1	11.87	2
Q8R2X0	EH-domain containing 2	11.83	3
Q62429	Tap1	11.79	3
B9EHR5	Unconventional myosin-VIIb	11.75	2
Q8CFH0	Prolactin-like protein C 3, isoform CRA_a	11.74	2
B2RWS3	Receptor transporter protein 3	11.71	2
E9Q926	Serine protease inhibitor A3M	11.66	2
Q6P5E8	Diacylglycerol kinase theta	11.63	2
Q3UFC6	Desmoglein 3	11.62	2
B9EKA3	Deleted in liver cancer 1	11.59	2
Q8K3W3	Cancer susceptibility candidate 3	11.58	2
O88829	Lactosylceramide alpha-2,3-sialyltransferase	11.58	2
Q8K0T4	Katanin p60 ATPase-containing subunit A-like 1	11.56	2
E9QN15	DNA-dependent protein kinase catalytic subunit	11.54	2
B1PSD9	cAMP-specific 3',5'-cyclic phosphodiesterase 4D	11.52	2
Q14B51	UDP-N-acetyl-alpha-D-galactosamine:polypeptide N-acetylgalactosaminyltransferase 5	11.50	2
Q99NE9	Pre-B-cell leukemia transcription factor 4	11.48	2
Q8BL66	Early endosome antigen 1	11.47	2
B2RS91	RNA polymerase I-specific transcription initiation factor RRN3	11.39	2
Q2T9H5	Vanin 3	11.39	3
B2RWX0	Myosin, heavy polypeptide 1, skeletal muscle, adult	11.36	2
A2AN08	E3 ubiquitin-protein ligase UBR4	11.29	2
Q920Q2	DNA repair protein REV1	11.28	2
B1AQ77	Keratin 15, isoform CRA_a	11.23	2
Q7TRI0	Olfactory receptor 790	11.23	4
Q8K0C6	Kallikrein 1-related peptidase b8	11.20	2
B7ZP32	Zinc finger protein 408	11.17	2
E9QLV2	Dopey-2	11.17	2
Q99PL6	UBX domain-containing protein 6	11.17	2
B2RUG8	Additional sex combs like 2 (Drosophila)	11.16	2
B7ZW99	Formin 1	11.14	2
Q6P3A4	V-set and immunoglobulin domain-containing protein 8	11.13	5
Q99PT1	Rho GDP-dissociation inhibitor 1	11.11	2
Q6PHC1	Enolase 1	11.05	2

B2RT41	Zinc finger, C3H1-type containing	11.04	2
A5X3G4	Kinase suppressor of ras 2	11.03	2
E9QMG5	Sorting nexin-25	11.02	2
P97298	Pigment epithelium-derived factor	11.00	6
Q3TLN1	Peroxisomal biogenesis factor 12, isoform CRA_a	10.95	2
Q2TBA4	Basonuclin 2	10.91	2
A2ADZ8	IQ domain-containing protein C	10.90	2
Q68FM6	Leucine rich repeat and fibronectin type III, extracellular 2	10.89	2
Q3TFK5	G-patch domain-containing protein 4	10.87	2
Q5RKV3	Phosphoglycerate kinase 2	10.82	2
Q05DV1	NADPH--cytochrome P450 reductase	10.78	2
Q155P7	Leucine, glutamic acid, lysine family 1 protein	10.75	2
Q8C1E7	Transmembrane protein 120A	10.71	3
B7ZN53	Armadillo repeat containing 3	10.68	2
B7NZ86	Beta-1,4-galactosyltransferase	10.65	2
B9EIZ9	NLR family, pyrin domain containing 9B	10.62	2
Q9JL95	Proteoglycan 3	10.60	2
P48036	Annexin A5	10.57	2
Q8CD92	Tetratricopeptide repeat protein 27	10.54	2
Q9CWY3	N-lysine methyltransferase SETD6	10.54	2
Q9ES82	Popeye domain-containing protein 2	10.54	2
A2ALD8	LIM homeobox protein 3	10.52	2
Q8BH59	Calcium-binding mitochondrial carrier protein Aralar1	10.52	4
Q3UKC1	Tax1-binding protein 1 homolog	10.51	2
Q8K2Q9	Shootin-1	10.50	3
Q3V036	Coiled-coil domain-containing protein 27	10.49	3
Q7TML3	Solute carrier family 35 member F2	10.47	2
Q91YX0	Thymocyte selection associated family member 2	10.43	3
Q3THK7	GMP synthase [glutamine-hydrolyzing]	10.41	2
Q6P9J5	KN motif and ankyrin repeat domain-containing protein 4	10.39	3
B2RXR2	Pik3r4	10.36	2
B2RXT8	AT rich interactive domain 4A (RBP1-like)	10.35	2
Q8R420	ATP-binding cassette sub-family A member 3	10.27	3
A9C437	Chloride channel protein 2	10.24	2
A6PWV7	Lysosomal amino acid transporter 1 homolog	10.22	2
E9QLS8	ATR-interacting protein	10.20	2
B2RY59	Neuralized homolog 4 (Drosophila)	10.19	2
Q91VJ2	Protein kinase C delta-binding protein	10.19	3
Q05D44	Eukaryotic translation initiation factor 5B	10.18	2
Q0P520	NLR family, pyrin domain containing 4C	10.14	2
Q8BFX3	BTB/POZ domain-containing protein KCTD3	10.12	2
Q01237	3-hydroxy-3-methylglutaryl-coenzyme A reductase	10.11	2
Q3UQU0	Bromodomain-containing protein 9	10.10	2
Q6PGC1	ATP-dependent RNA helicase Dhx29	10.09	4
A2AL17	Rho GTPase-activating protein 11A	10.07	2
Q14DK8	Phospholipase A2, group IVD	10.06	3
Q7TS23	Olfactory receptor 251	10.06	3
Q3UPP8	Centrosomal protein of 63 kDa	10.02	2
Q5SV85	Synergin gamma	10.01	5

Q8R3C6	RNA binding motif protein 19	10.00	2
Q8K3V4	Protein-arginine deiminase type-6	9.94	2
Q61982	Neurogenic locus notch homolog protein 3	9.93	4
Q0P678	Zinc finger CCCH domain-containing protein 18	9.91	3
P68368	Tubulin alpha-4A chain	9.86	2
Q66JW2	Golgi autoantigen, golgin subfamily a, 1	9.86	2
Q8R1T2	Cullin 4A	9.84	2
A9P6P9	Testis-specific serine/threonine protein kinase 5 variant delta	9.78	2
Q9QZA0	Carbonic anhydrase 5B, mitochondrial	9.74	2
O88990	Alpha-actinin-3	9.71	2
D5G1T0	Ankyrin repeat domain 33, isoform CRA_c	9.68	2
Q0P543	Gastric inhibitory polypeptide receptor	9.65	2
A8DUL5	Hemoglobin, beta adult major chain	9.62	2
Q8BW49	Tetratricopeptide repeat protein 12	9.62	3
A8DUL5	Hemoglobin, beta adult major chain	9.62	2
P03995	Glial fibrillary acidic protein	9.62	2
B7ZWK2	Myotubularin-related protein 5	9.61	2
Q67E05	Bactericidal permeability-increasing protein	9.61	6
Q6P5U7	Kiaa1239	9.59	6
A2SW42	Zinc finger protein 462	9.57	2
Q3THE6	Ferritin	9.57	2
B2RT12	Ubiquitin specific peptidase 37	9.52	2
Q9CQT1	Methylthioribose-1-phosphate isomerase	9.52	2
C0LQ88	Syncoilin isoform 3	9.48	2
Q7TS72	Inositol-trisphosphate 3-kinase C	9.47	2
Q920R0	Amyotrophic lateral sclerosis 2 (juvenile)	9.45	3
Q6PIJ4	Nuclear factor related to kappa-B-binding protein	9.40	2
E9QM11	Microtubule-associated protein 1B	9.35	2
Q6PCP7	Probable G-protein coupled receptor 156	9.34	2
Q6PDF3	SV2 related protein homolog (rat)-like	9.31	3
Q8K2I4	Beta-mannosidase	9.31	3
Q9D2L5	Inactive carboxypeptidase-like protein X2	9.31	2
Q8BGV8	Mitochondrial dynamic protein MID51	9.29	2
P59111	Potassium voltage-gated channel subfamily H member 8	9.27	2
Q14DP5	Catenin (Cadherin associated protein), alpha 3	9.27	3
Q5DTI8	Extended synaptotagmin-3	9.27	3
E9QKD7	Dynein heavy chain 5, axonemal	9.25	2
A2AKB9	DDB1- and CUL4-associated factor 10	9.23	2
Q9QZ67	Protein phosphatase 1D	9.23	2
**Q62059**	**Versican core protein**	**9.20**	**2**
O08850	Regulator of G-protein signaling 5	9.18	2
Q8C3L1	Ssu-2 homolog (C. elegans)	9.16	3
O88783	Coagulation factor V	9.16	2
B7ZNF8	Chromodomain helicase DNA binding protein 9	9.15	2
A6H5W2	Coiled-coil domain-containing protein 87	9.13	2
**P11087**	**Collagen alpha-1(I) chain**	**9.11**	**2**
Q64092	Transcription factor E3	9.09	3
A2AJG1	Acyl-CoA-binding domain-containing protein 7	9.08	2
D6CHX5	Hepatoma-derived growth factor-related protein 2	9.06	2

E9QL35	Alpha-kinase 2	9.06	2
Q80ZJ6	Zyg-11 related, cell cycle regulator	9.02	2
Q497V3	La ribonucleoprotein domain family, member 2	8.96	3
Q9WVA4	Transgelin-2	8.94	2
Q9WTU0	Lysine-specific demethylase PHF2	8.92	2
P28184	Metallothionein-3	8.90	2
Q5SXY1	Cytospin-B	8.86	2
Q62504	Msx2-interacting protein	8.86	2
B9EHV0	Leucine rich repeat containing 7	8.84	2
O54984	ArsA arsenite transporter, ATP-binding, homolog 1 (bacterial)	8.84	2
P62897	Cytochrome c, somatic	8.84	2
E9QM01	E3 SUMO-protein ligase RanBP2	8.82	2
Q8K448	ATP-binding cassette sub-family A member 5	8.81	2
Q9R269	Periplakin	8.81	2
B7ZWG4	Tripartite motif-containing 40	8.77	2
Q8K0Z5	Tropomyosin 3, gamma	8.74	2
B5LBC3	RAD51D transcript variant delta 9,10	8.73	2
Q6P8I3	WD repeat domain 70	8.72	3
Q7TS74	Cytoskeleton-associated protein 2-like	8.72	4
Q8BNJ3	Nicotinamide nucleotide adenylyltransferase 2	8.72	2
Q5SUR0	Phosphoribosylformylglycinamidine synthase	8.68	2
Q505K2	Family with sequence similarity 160, member A1	8.67	4
D3Z1J2	Coiled-coil domain-containing protein 112	8.65	3
Q8R4G5	Truncated N-acetylglucosaminyltransferase V	8.64	3
B1ARH0	Hypermethylated in cancer 1 protein	8.59	2
Q059Q1	WD repeat-containing protein 7	8.55	2
C6EQH1	ASL1/Fgf17 fusion protein	8.54	2
Q9JI58	Retinoic acid early-inducible protein 1-delta	8.54	2
P70670	Naca	8.53	2
Q8VC03	Echinoderm microtubule-associated protein-like 3	8.51	2
Q99N99	3-oxo-5-alpha-steroid 4-dehydrogenase 2	8.49	2
E9QPW5	Kinesin-like protein KIF20B	8.48	2
P70351	Histone-lysine N-methyltransferase EZH1	8.47	4
Q8K0M3	Sorbin and SH3 domain containing 3	8.44	3
A2RRJ0	Desmoglein-2	8.42	2
B2RTP7	Keratin 2	8.41	3
Q8CGD2	Cysteine-rich secretory protein LCCL domain-containing 1	8.40	2
Q9WUU9	80 kDa MCM3-associated protein	8.39	2
Q80W71	Pleckstrin homology domain-containing family A member 8	8.39	4
Q149S1	Tektin 4	8.38	2
Q8C3Y4	Kinetochore-associated protein 1	8.38	3
Q8CJF7	AT hook containing transcription factor 1	8.38	3
Q9JHD1	Histone acetyltransferase KAT2B	8.38	2
B7ZWL7	Leucine rich repeat containing 36	8.36	2
P53569	CCAAT/enhancer-binding protein zeta	8.33	3
Q6DIB5	Multiple epidermal growth factor-like domains protein 10	8.30	2
Q08EK4	Keratin 77	8.29	2
Q01853	Transitional endoplasmic reticulum ATPase	8.28	2
Q9Z1W9	STE20/SPS1-related proline-alanine-rich protein kinase	8.27	2

Q9ESC5	GTPase ERA-W	8.24	2
Q62392	Pleckstrin homology-like domain family A member 1	8.19	2
B9EIW5	Nuclear receptor coactivator 7	8.17	2
Q920P3	Deleted in bladder cancer protein 1 homolog	8.17	3
Q8R2C5	Vomeronasal receptor V1RC29	8.17	2
O88623	Ubiquitin carboxyl-terminal hydrolase 2	8.13	2
Q5SWY7	Family with sequence similarity 83, member G	8.13	3
B7ZP07	Ubiquitin specific peptidase, pseudogene (USP17 homolog)	8.10	2
B9EIV3	Calcium channel, voltage-dependent, alpha 2/delta subunit 4	8.09	2
Q8R4N0	Citrate lyase subunit beta-like protein, mitochondrial	8.08	3
Q8VG71	Olfactory receptor 907	8.08	2
Q8VDE8	Nuclear receptor co-repressor 1	8.07	2
B2RWW0	T-cell activation Rho GTPase-activating protein	8.05	2
Q5SSZ5	Tensin-3	8.03	3
B7ZNF6	Catenin (cadherin associated protein), delta 2	7.96	2
Q80XT6	Lipin 1	7.96	3
Q8K4L2	Supervillin	7.93	3
Q8K4L2	Supervillin	7.93	3
Q61115	Patched homolog 1	7.91	2
C5H7S5	Vascular endothelial growth factor receptor 2	7.90	2
Q8VIN7	Cardiac triadin isoform 3	7.90	3
B1AR51	Dynein, axonemal, heavy chain 9	7.88	2
Q61903	Myeloid secondary granule protein	7.87	3
Q8VDN9	Coiled-coil domain containing 21	7.83	3
Q8BHI4	Kelch repeat and BTB domain-containing protein 3	7.76	2
B2RXW8	Ppfia1 protein	7.74	2
P62192	26S protease regulatory subunit 4	7.73	2
Q3ZAT5	Onecut2 protein	7.73	2
Q3ZAT5	Onecut2 protein	7.73	2
Q8BGU5	Cyclin-Y	7.72	2
B2RXA1	Plcxd2	7.69	2
Q61769	Ki-67 protein	7.69	2
Q09XV5	Chromodomain-helicase-DNA-binding protein 8	7.67	2
Q00941	GM-CSF-R-alpha	7.66	3
Q7M6Z4	Kinesin-like protein KIF27	7.66	3
A0JNY3	Gephyrin	7.65	2
Q8CDC7	Zinc finger and BTB domain-containing protein 9	7.64	2
Q9WV35	Probable C->U-editing enzyme APOBEC-2	7.59	2
P09066	Homeobox protein engrailed-2	7.53	2
Q499F8	Zinc finger protein 445	7.52	3
Q7TMQ7	WD repeat-containing protein 91	7.52	3
Q8VHQ2	Suppressor of cytokine signaling 7	7.52	3
Q8VI46	Canalicular multispecific organic anion transporter 1	7.51	3
B2RY84	Kinesin family member 14	7.47	3
A5J0L5	Interleukin 21	7.47	2
Q2TAW4	Myosin, heavy polypeptide 6, cardiac muscle, alpha	7.42	3
Q8VGG7	Olfactory receptor 1282	7.40	3
Q75WC0	DNA polymerase subunit gamma-1	7.35	3
Q922Y0	Dual specificity tyrosine-phosphorylation-regulated kinase 3	7.34	2

Q8CH25	SAFB-like transcription modulator	7.32	3
Q497I5	Late cornified envelope 3A	7.31	3
Q9D8S3	ADP-ribosylation factor GTPase-activating protein 3	7.31	2
B2RY58	Hyperpolarization-activated, cyclic nucleotide-gated K+ 4	7.30	3
Q9JI44	DNA methyltransferase 1-associated protein 1	7.24	2
Q8K566	Cysteine sulfinic acid decarboxylase	7.23	3
A2AQ50	Solute carrier family 12 member 1	7.22	2
P43142	G-protein coupled receptor 182	7.21	2
B7ZWG5	Coiled-coil domain containing 60	7.20	2
Q6P5H2	Nestin	7.20	3
Q8BWF0	Succinate-semialdehyde dehydrogenase, mitochondrial	7.19	4
B7ZNX6	Erbb2 interacting protein	7.17	2
Q14CH0	Family with sequence similarity 171, member B	7.17	3
Q3U319	E3 ubiquitin-protein ligase BRE1B	7.14	2
Q924W5	Structural maintenance of chromosomes protein 6	7.11	2
Q99JX6	Annexin A6	7.10	2
Q9CRT8	Exportin-T	7.08	2
A2BGH0	BPI fold-containing family B member 4	7.07	2
B2RXZ4	Kcn10a	7.00	2
B2RY11	ATP-binding cassette, sub-family A (ABC1), member 12	7.00	2
E9QPI0	HEAT repeat-containing protein 5A	7.00	2
Q7M6Z8	Kinesin light chain 1F	6.99	3
Q8R1Q3	Angiopoietin-related protein 7	6.96	2
Q8VGD0	Olfactory receptor 25	6.96	2
Q5DTY9	BTB/POZ domain-containing protein KCTD16	6.93	2
Q9WU22	Tyrosine-protein phosphatase non-receptor type 4	6.92	2
P0C5J4	Probable G-protein coupled receptor 52	6.91	2
Q5SSG4	Growth arrest-specific 2 like 1	6.91	2
Q9ER73	Elongator complex protein 4	6.91	2
**Q62234**	**Myomesin-1**	**6.90**	**2**
Q3MI48	Junctional sarcoplasmic reticulum protein 1	6.88	2
B9EKE4	Periphilin 1	6.87	2
Q6R5P3	Zinc finger protein 677	6.84	4
Q8N7N5	DDB1- and CUL4-associated factor 8	6.84	2
Q91Z67	SLIT-ROBO Rho GTPase-activating protein 2	6.83	2
Q7TNB8	Strawberry notch homolog 2	6.80	2
Q4VAA2	Carnitine deficiency-associated gene expressed in ventricle 3	6.79	3
Q8K087	G-protein coupled receptor 1	6.79	2
Q3URU2	Paternally-expressed gene 3 protein	6.78	2
Q80YP3	Chromosome-associated kinesin KIF4	6.77	2
Q80U57	Regulating synaptic membrane exocytosis protein 3	6.77	4
**Q3UXK8**	**Biglycan**	**6.76**	**2**
Q91YD6	Villin-like protein	6.76	3
Q80W93	Hydrocephalus-inducing protein	6.75	2
B9EJA6	Tubulin tyrosine ligase-like family, member 6	6.74	2
Q61193	Ral guanine nucleotide dissociation stimulator-like 2	6.73	3
C3S7Q6	Ojoplano variant B	6.71	2
Q059P4	Filamin A interacting protein 1	6.71	2
Q76HP3	Transmembrane protein 132D	6.70	4

Q62000	Mimecan	6.69	5
Q14C50	Solute carrier family 27 (Fatty acid transporter), member 6	6.66	3
Q52KG5	Kinesin-like protein KIF26A	6.66	3
Q9CX00	Increased sodium tolerance 1 homolog (yeast)	6.65	2
Q3B807	Transcription elongation regulator 1-like protein	6.63	2
B7ZMT5	Multimerin 1	6.60	2
E9QLR6	Serine/threonine-protein kinase SMG1	6.60	2
Q9DBD2	E3 ubiquitin-protein ligase MARCH8	6.60	2
Q0VGI9	Gata5 protein	6.58	2
B7ZNW9	Slit homolog 2 (Drosophila)	6.55	2
A1L343	Transglutinase 3	6.51	2
B2RU69	CUB and zona pellucida-like domain-containing protein 1	6.51	2
Q8BML1	Protein-methionine sulfoxide oxidase MICAL2	6.49	3
B7ZNK9	Spermatogenesis associated 5	6.48	2
B2RWX1	Rho GTPase activating protein 21	6.47	2
Q91W97	Hexokinase domain containing 1	6.47	3
Q9EQW6	Oligodendrocyte transcription factor 2	6.47	2
B9EJ52	ATPase, class V, type 10D	6.46	2
E2IUN6	Shugoshin-like 2B	6.45	2
B2RXY1	Spock1	6.42	2
Q05A56	Hyaluronidase-4	6.40	2
Q8BXV2	Bri3 binding protein	6.40	3
Q8CI43	Myosin light chain 6B	6.39	3
D3Z0W5	Inactive phospholipase C-like protein 1	6.38	2
Q8CGY8	Ogt	6.36	2
Q6PDB6	Prolactin-2C3	6.34	4
Q76LL6	FH1/FH2 domain-containing protein 3	6.33	2
P56384	ATP synthase lipid-binding protein, mitochondrial	6.32	3
Q8C0S1	DIS3 mitotic control homolog (S. cerevisiae)-like	6.31	2
B2RUG6	Dedicator of cytokinesis 4	6.30	2
Q6PFX2	BEN domain-containing protein 6	6.26	2
Q5SX39	Myosin-4	6.26	3
B7ZND5	Purinergic receptor P2X, ligand-gated ion channel, 1	6.23	2
A2AIM4	Tropomyosin beta chain	6.21	2
P47856	Glutamine fructose-6-phosphate amidotransferase 1	6.21	2
A2ALI5	Adherens junction-associated protein 1	6.20	2
Q8QZR5	Alanine aminotransferase 1	6.17	3
Q921I6	SH3 domain-binding protein 4	6.14	2
P47810	Wee1-like protein kinase	6.11	2
Q571C7	Transcription factor TFIIIB component B'' homolog	6.11	2
Q7TNU9	Regulator of G-protein signaling 16	6.09	5
Q9EST3	Eukaryotic translation initiation factor 4E transporter	6.09	2
Q7TSZ1	Xeroderma pigmentosum, complementation group C	6.09	4
E9QNJ2	Leucine-rich repeat serine/threonine-protein kinase 2	6.06	2
Q7M710	Taste receptor type 2 member 125	6.05	3
Q5ISE2	Zinc finger protein 36, C3H1 type-like 3	6.04	2
Q8R0Z4	Sorting nexin 6	6.04	3
Q5ISE2	Zinc finger protein 36, C3H1 type-like 3	6.04	2
Q8CDK3	IQ and ubiquitin-like domain-containing protein	6.02	3

B1ATT5	Ketosamine-3-kinase	6.01	2
B2KFW1	Zinc finger and SCAN domain-containing protein 20	6.01	2
P60867	40S ribosomal protein S20	6.01	2
Q3U452	ATP synthase subunit alpha	6.01	2
Q3V1I0	Lysozyme g-like protein 2	5.99	3
Q8BI72	CDKN2A-interacting protein	5.95	2
Q8BKY8	mTERF domain-containing protein 3, mitochondrial	5.95	2
E9QKZ9	Proline-rich transmembrane protein 4	5.94	2
D3Z7P3	Glutaminase kidney isoform, mitochondrial	5.94	2
Q8K2G3	DiGeorge syndrome critical region gene 2	5.93	3
Q148W1	Solute carrier organic anion transporter family, member 5A1	5.91	2
Q8K2N9	Annexin A8	5.91	2
A3KGI4	Cancer susceptibility candidate 5	5.89	2
**Q61221**	**Hypoxia-inducible factor 1-alpha**	**5.77**	**3**
Q80U56	Late secretory pathway protein AVL9 homolog	5.75	3
Q6ZQH8	Nucleoporin NUP188 homolog	5.74	5
Q8R4E6	Purine-rich element-binding protein gamma	5.74	2
Q9QZ83	Gamma actin-like protein	5.72	2
Q8BKT2	Transcription factor HES-7	5.70	4
B2RPV0	Dmrta1	5.69	2
B2RXX1	Centrosomal protein 110	5.69	2
P70277	Alpha-N-acetylgalactosaminide alpha-2,6-sialyltransferase 2	5.68	2
B2RUJ4	ATPase family, AAA domain containing 5	5.67	2
Q8BL06	Inactive ubiquitin carboxyl-terminal hydrolase 54	5.67	3
Q9EPU0	Regulator of nonsense transcripts 1	5.67	2
A7XUZ6	Selection and upkeep of intraepithelial T-cells protein 6	5.67	2
B1AWS8	Angiopoietin-1 receptor	5.65	2
A2AM05	Centlein	5.64	2
Q05D38	RAB27b, member RAS oncogene family	5.63	2
Q9Z247	Peptidyl-prolyl cis-trans isomerase FKBP9	5.63	2
Q7M6Y6	Maestro heat-like repeat-containing protein family member 2B	5.62	4
Q640L5	Coiled-coil domain-containing protein 18	5.60	3
B2RY18	FERM and PDZ domain containing 1	5.58	2
Q0V930	Talin 1	5.58	2
Q80ZQ0	Sperm acrosome membrane-associated protein 4	5.58	4
Q0VGQ5	PI3K-alpha	5.57	3
B2RWS7	Zinc finger protein 609	5.54	2
F1ABR9	Ager	5.54	2
B6ZCE5	Proteinase 3	5.54	2
Q3KPB0	Olfactory receptor 616	5.54	2
Q8JZU4	Forkhead box A3	5.53	2
D3Z6S9	Family with sequence similarity 194, member A	5.52	2
Q148W8	Inactive dual specificity phosphatase 27	5.52	2
B4XVN8	SET domain bifurcated protein 2 variant A.1	5.51	2
P16015	Carbonic anhydrase 3	5.51	2
Q8BIW9	Chromosome transmission fidelity protein 18 homolog	5.49	4
A1L3S7	Gatad2b	5.48	2
Q149F3	Gspt2	5.45	2
Q64163	Transcription factor Dp-2	5.45	2

Q8VED5	Keratin, type II cytoskeletal 79	5.41	2
Q7TPR0	E3 ubiquitin-protein ligase RNF14	5.40	3
Q8CCP0	Nuclear export mediator factor	5.38	2
Q5D0D8	Glucosaminyl (N-acetyl) transferase 1, core 2	5.36	2
Q6PB60	Basic helix-loop-helix domain containing, class B9	5.36	2
Q9CWR8	DNA (cytosine-5)-methyltransferase 3-like	5.35	2
**E9Q6U9**	**Collagen alpha-2(I) chain**	**5.35**	**3**
Q62381	Tolloid-like protein 1	5.33	7
Q91Y06	Protocadherin beta 13	5.33	2
Q8CG46	Structural maintenance of chromosomes protein 5	5.33	2
Q52KI9	Zinc finger protein 592	5.32	2
Q5DTM8	E3 ubiquitin-protein ligase BRE1A	5.31	2
Q6NS59	Family with sequence similarity 135, member A	5.30	2
Q99LG0	Ubiquitin carboxyl-terminal hydrolase 16	5.30	2
A2AQ19	RNA polymerase-associated protein RTF1 homolog	5.27	2
B2RWU4	Cordon-bleu	5.27	2
B2RWU4	Zinc finger protein 609	5.27	2
B7ZMU4	Rho-related BTB domain containing 1	5.23	2
Q3LRV6	Voltage-gated chloride channel ClC-4A	5.22	2
A2AA59	PHD Finger protein 15	5.21	2
D3YYK0	Abhydrolase domain containing 11, isoform CRA_b	5.21	3
P50396	Rab GDP dissociation inhibitor alpha	5.21	2
Q6IRU2	Tropomyosin alpha-4 chain	5.20	2
Q8CHE4	Phlpp1	5.19	3
Q8JZQ3	ST8 alpha-N-acetyl-neuraminide alpha-2,8-sialyltransferase 5	5.18	2
Q9D2I5	LisH domain-containing protein ARMC9	5.17	2
Q99K58	Fibulin 2	5.14	3
Q4FZC9	Nesprin-3	5.13	2
A2RT18	Testis expressed gene 16	5.12	2
Q14BE5	Forkhead box B2	5.12	2
Q99J95	Cyclin-dependent kinase 9	5.12	2
A2A6H3	F-box only protein 47	5.11	2
Q8BVN3	Cation channel sperm-associated protein 4	5.06	2
Q6DIC7	Phosphatidylinositol 4-kinase, catalytic, alpha polypeptide	5.04	3
**P19001**	**Keratin, type I cytoskeletal 19**	**5.00**	**2**
Q05AH2	Zinc finger, CW type with PWWP domain 2	4.99	2
Q64433	10 kDa heat shock protein, mitochondrial	4.99	7
Q7TQU4	Olfactory receptor 1361	4.99	3
P09027	Homeobox protein Hox-D3	4.97	2
Q05BG1	CCR4-NOT transcription complex, subunit 4	4.96	2
E9QKT8	Alstrom syndrome protein 1 homolog	4.93	2
**P28654**	**Decorin**	**4.93**	**2**
B2RWX5	Centrosomal protein 250	4.91	2
Q5F204	Putative malate dehydrogenase 1B	4.91	8
Q8VI16	B3gnt9	4.88	2
Q2KHP0	Kcna1	4.87	3
E9QNC3	Serine/threonine-protein kinase Nek11	4.86	2
Q9D3D0	Alpha-tocopherol transfer protein-like	4.85	2
P08905	Lysozyme C-2	4.85	2

Q0VGB8	Synaptic nuclear envelope 1	4.83	2
Q8VDU9	Tripartite motif containing 41	4.82	3
B2RRD1	Tudor domain containing 6	4.81	2
Q9JK37	Myozenin-1	4.80	2
Q14AP0	Thioredoxin domain containing 8	4.78	2
Q8C120	SH3 domain-containing RING finger protein 3	4.77	2
Q9WVS5	Chaperonin containing TCP-1 theta subunit	4.77	2
Q0VDP6	Hypocretin (orexin) receptor 1	4.76	3
Q78HU3	Multivesicular body subunit 12A	4.74	2
Q9DD20	Methyltransferase-like protein 7B	4.71	2
E9QJZ4	Fibrillin-2	4.69	2
B9EKK3	IQ motif containing GTPase activating protein 2	4.68	2
P00920	Carbonic anhydrase 2	4.67	2
E9QKV8	Nrde-2 necessary for RNA interference, domain containing	4.65	3
Q3V3F1	Nicotinamide mononucleotide adenylyltransferase 3	4.65	2
A2BE28	Ribosomal biogenesis protein LAS1L	4.64	2
B7ZP22	Heterogeneous nuclear ribonucleoprotein A2/B1	4.64	2
B2RWX4	Rint1	4.63	2
Q05BH2	Tetraspanin-5	4.63	3
B1AXY5	Beta-1,4-galactosyltransferase 1	4.62	2
**P11679**	**Keratin, type II cytoskeletal 8**	**4.62**	**2**
Q75WD0	Protein-arginine deiminase type-2	4.61	3
P28665	Murinoglobulin-1	4.59	2
Q80TA6	Myotubularin-related protein 12	4.58	3
P59530	Taste receptor type 2 member 7	4.58	2
P51150	Ras-related protein Rab-7a	4.57	2
Q68EF9	Signal peptide, CUB domain, EGF-like 1	4.56	2
D5MP63	Kinesin-like protein KIF26B	4.55	2
B7FAU9	Filamin, alpha	4.54	2
A1A557	Cartilage oligomeric matrix protein	4.52	2
E9QMY2	E3 ubiquitin-protein ligase CBL-B	4.50	2
Q7TNT9	Elk3	4.50	3
C4P6S0	Sperm head and tail associated protein	4.49	2
Q9JJJ0	Calcium transporting protein homolog	4.49	2
Q6PBG2	NADH dehydrogenase (ubiquinone) 1 alpha subcomplex 11	4.47	2
E9Q010	Dynein heavy chain 8, axonemal	4.46	4
Q6SLK2	Protein kinase lysine deficient 1	4.42	4
B2RXT5	Glucose-6-phosphate isomerase	4.39	2
O55124	Myomesin 2	4.38	2
Q62261	Spectrin beta chain, non-erythrocytic 1	4.37	4
O88329	Unconventional myosin-Ia	4.36	2
Q3TIU4	2',5'-phosphodiesterase 12	4.35	2
E3VWA1	Slc4a4	4.31	2
Q8R2H9	Phosphatase, orphan 1	4.31	2
Q9CSH3	Exosome complex exonuclease RRP44	4.30	2
Q810R0	Sycp3 like Y-linked	4.28	4
O70251	Elongation factor 1-beta	4.27	2
A2AHK7	Mucin-15	4.26	2
B2RXX7	Nance-Horan syndrome	4.25	2

E9QM71	Cytoplasmic dynein 1 heavy chain 1	4.24	2
Q8BFZ3	Actin, beta-like 2	4.24	3
Q04899	Cyclin-dependent kinase 18	4.22	3
Q0VB79	T-cell ecto-ADP-ribosyltransferase 1	4.21	2
Q9EQD6	Keratin intermediate filament 16a	4.20	2
Q2WF71	Lrfn1	4.20	2
B8XCJ6	Protein unc-80 homolog	4.18	2
Q6P9Q2	Polymerase (RNA) I polypeptide B	4.18	5
Q9CVL5	CutA divalent cation tolerance homolog	4.16	2
O88799	Zonadhesin	4.15	2
Q9EPL8	Importin-7	4.14	2
Q1ZZX0	Toll-like receptor 5	4.13	2
Q8CIP4	MAP/microtubule affinity-regulating kinase 4	4.13	3
Q9EPS7	Pheromone receptor V3R6	4.13	2
Q921T1	Transforming growth factor, beta 2	4.12	2
E9PZZ1	PR domain zinc finger protein 13	4.10	2
Q2XP45	Phosphatidic acid-preferring phospholipase A1 variant 2	4.10	2
E9QMR9	Protocadherin Fat 4	4.07	2
Q91ZF5	Calcium-activated chloride channel CLCA4	4.06	2
P27573	Myelin protein P0	4.06	2
Q99P72	Reticulon-4	4.06	2
B7ZMZ7	DIP2 disco-interacting protein 2 homolog C (Drosophila)	4.04	2
Q91WB5	Androgen binding protein alpha	4.04	2
Q8C963	Coiled-coil domain-containing protein 159	4.01	2
Q8K0J2	B3gnt7	4.01	2
Q8VDV3	Guanine nucleotide exchange factor for Rab-3A	4.01	3
Q8K287	Armadillo repeat containing 5	4.00	2
Q05DC5	Mitochondrial ribosomal protein L3	3.99	2
E9PV27	Pheromone receptor V3R1	3.96	2
Q924J9	Six-transmembrane epithelial antigen of the prostate	3.94	2
B7ZWK3	Spectrin alpha, non-erythrocytic 1	3.89	2
C6EQH2	ASL1/Herc2 fusion protein	3.88	2
P04104	Keratin, type II cytoskeletal 1	3.88	2
P62878	E3 ubiquitin-protein ligase RBX1	3.88	2
B7ZNM9	DNA topoisomerase 1, mitochondrial	3.86	2
Q8R429	Sarcoplasmic/endoplasmic reticulum calcium ATPase 1	3.86	2
B2RQL6	Myosin light chain kinase 3	3.85	2
A2ADF7	Solute carrier family 25 member 34	3.82	2
Q9D2X5	MAU2 chromatid cohesion factor homolog	3.80	2
Q99L13	3-hydroxyisobutyrate dehydrogenase, mitochondrial	3.77	3
Q9JI38	tRNA pseudouridine(38/39) synthase	3.77	2
Q4VBF8	Signal-induced proliferation-associated 1 like 1	3.76	3
Q8C0P0	Microtubule associated serine/threonine kinase-like	3.76	4
E9QAC1	Bromodomain and WD repeat-containing protein 1	3.75	3
Q8R1X8	Oxysterol-binding protein 6	3.75	2
A0AUM9	Translation initiation factor eIF-2B subunit gamma	3.75	2
B1ARM7	Aspm1	3.75	2
A6NAW3	Alpha46-takusan	3.73	2
D3YZF7	V-set and immunoglobulin domain-containing protein 10-like	3.72	4

E3VVQ8	Supervillin muscle-specific isoform	3.72	2
Q6NS69	APC membrane recruitment protein 3	3.72	5
B7ZWN0	Sodium channel, voltage-gated, type IX, alpha	3.69	2
Q0VBT4	Tryptophan 5-hydroxylase 2	3.66	2
B2RY47	Rhotekin 2	3.65	2
B2RXR3	CCR4-NOT transcription complex, subunit 1	3.64	2
Q1XGY5	Fras1 related extracellular matrix protein 2	3.62	3
Q8K203	Endonuclease 8-like 3	3.62	3
Q9JK98	Clusterin	3.62	2
**Q61282**	**Aggrecan core protein**	**3.62**	**2**
Q922R7	Testis expressed gene 10	3.60	3
B2RSV7	FYVE, RhoGEF and PH domain containing 6	3.57	2
Q99MQ4	Asporin	3.57	3
B2RX10	Fanconi anemia, complementation group M	3.56	3
Q80UM4	Tyrosine-protein phosphatase non-receptor type 12	3.54	2
Q0QJG0	Nanog homeobox pseudogene	3.53	3
Q04447	Creatine kinase B-type	3.52	2
Q6NV59	Lysyl oxidase-like 4	3.48	2
Q8K0R1	Xinc finger protein 644	3.47	3
B1AWC9	Phosphodiesterase 4B, cAMP specific	3.45	2
B9UM22	G-protein-coupled receptor 81	3.45	2
Q03172	Zinc finger protein 40	3.45	3
Q8CFE8	F-box and WD-40 domain protein 17	3.45	3
Q148V8	Family with sequence similarity 83, member H	3.43	3
Q8CH77	Neuron navigator 1	3.43	2
C6EQK3	L1 unspliced fusion gene protein	3.42	2
A2AQ14	Protein Mis18-beta	3.41	2
P15533	Tripartite motif-containing protein 30A	3.36	2
B7ZN63	PRELI domain containing 2	3.33	2
P70124	Serpin B5	3.33	2
Q7M753	Pantothenate kinase 2	3.32	3
Q8BHL3	TBC1 domain family member 10B	3.32	3
Q7TQ32	Hemojuvelin	3.32	2
Q9CQ52	Chymotrypsin-like elastase family member 3B	3.31	3
Q9CQ52	Chymotrypsin-like elastase family member 3B	3.31	3
B7ZNT3	Signal-regulatory protein beta 1C	3.30	2
B8JJ87	Apoptotic chromatin condensation inducer in the nucleus	3.30	2
O35452	Tenascin X	3.30	2
B8JJ87	Apoptotic chromatin condensation inducer in the nucleus	3.30	2
B2RY09	Enhancer trap locus 4	3.29	2
Q6GUA3	Periostin, osteoblast specific factor	3.29	2
B2RY09	Enhancer trap locus 4	3.29	2
B9EKA5	Melanophilin	3.28	2
Q7TMG7	Natriuretic peptide receptor 3	3.28	3
E9QLA1	Adamts20	3.26	2
Q9CR78	Myb/SANT-like DNA-binding domain-containing protein 3	3.24	2
Q91X22	Serpina1b	3.20	3
P43274	Histone H1.4	3.19	4
B7SNM9	Transformed mouse 3T3 cell double minute 1	3.15	2

P08399	Putative per-hexamer repeat protein 5	3.15	2
B7SNM9	Transformed mouse 3T3 cell double minute 1	3.15	2
B2RSU6	Cingulin-like 1	3.14	2
B2RXM4	Alpha N-terminal protein methyltransferase 1B	3.12	2
Q5F259	Ankyrin repeat domain-containing protein 13B	3.10	2
Q58IU7	Cathepsin R	3.07	2
Q91ZQ1	cGMP phosphodiesterase 6C	3.07	3
B2RQR2	Multiple PDZ domain protein	3.06	2
D6RGA8	Coenzyme Q2 homolog, prenyltransferase (yeast)	3.06	2
P63058	Thyroid hormone receptor alpha	3.04	2
B2RRJ9	Adenylate cyclase type 10	3.02	2
A2AFK4	GTP binding protein 5, isoform CRA_a	3.01	2
B9EKT6	IQ motif containing GTPase activating protein 3	3.01	2
P07724	Serum albumin	3.01	2
P51612	DNA repair protein complementing XP-C cells homolog	3.00	2
A2ARZ3	Fibrous sheath-interacting protein 2	2.99	2
Q9CWJ9	Bifunctional purine biosynthesis protein PURH	2.98	2
Q61584	Fragile X mental retardation syndrome-related protein 1	2.97	2
Q61584	Fragile X mental retardation syndrome-related protein 1	2.97	2
Q3TJG2	Acyl carrier protein	2.95	2
B2RUA0	Vomeronasal 1 receptor, I2	2.95	2
Q80ZS3	28S ribosomal protein S26, mitochondrial	2.93	3
Q8BZ71	SH3 and cysteine-rich domain-containing protein 3	2.93	2
P97401	Secreted frizzled-related protein 3	2.90	3
Q2NKY1	Thyroglobulin	2.90	3
P70196	TNF receptor-associated factor 6	2.88	4
Q8K4C5	Interleukin-17C	2.87	2
B7ZNH7	Collagen alpha-1(XIV) chain	2.86	2
Q8CFJ9	WD repeat-containing protein 24	2.86	3
P13542	Myosin-8	2.86	2
B1ATA2	CASK-interacting protein 2	2.84	2
Q0P5X1	Leucine-rich repeat and IQ domain-containing protein 1	2.83	2
Q0VFY6	Zinc finger, DHHC domain containing 17	2.82	3
Q80TR8	Vpr (HIV-1) binding protein	2.81	2
P51885	Lumican	2.80	2
Q9CXZ1	Ndufs4	2.80	2
Q6PG97	CD1d2	2.80	4
P08228	Superoxide dismutase [Cu-Zn]	2.78	2
A9Q751	Primary ciliary dyskinesia protein 1	2.76	2
B8QI35	Liprin-alpha 3	2.76	2
P09542	Myosin light chain 3	2.76	2
Q8C7H1	Methylmalonic aciduria type A homolog, mitochondrial	2.74	2
D3YXT2	ribosomal protein L10A, pseudogene 2	2.72	2
Q6PGN3	Serine/threonine-protein kinase DCLK2	2.72	4
Q6NXJ0	WW, C2 and coiled-coil domain containing 2	2.71	5
Q8R3C0	Mini-chromosome maintenance complex-binding protein	2.71	3
Q05BZ2	Poly (ADP-ribose) polymerase family, member 6	2.71	2
Q543V3	Insulin receptor substrate 1	2.71	4
Q8K1X1	WD repeat-containing protein 11	2.70	2

D3Z6F5	ATP synthase subunit alpha	2.68	2
D3Z6J1	DEP domain containing 1a, isoform CRA c	2.68	3
E9QNM2	Histone-lysine N-methyltransferase ASH1L	2.68	2
P68254	14-3-3 protein theta	2.68	2
Q5M956	BTB/POZ domain-containing protein KCTD1	2.68	2
B6VJS3	5-hydroxytryptamine receptor 7	2.67	2
C9K0Y7	AMPA-selective glutamate receptor 4 flop type	2.61	2
O35409	Glutamate carboxypeptidase 2	2.60	2
B7ZNV8	Ankyrin repeat domain 42	2.54	2
Q5SW50	Olfactory receptor 462	2.51	3
Q6PFZ4	Structural maintenance of chromosomes 3	2.50	3
Q8VCK3	Tubulin gamma-2 chain	2.49	3
Q9CR68	Cytochrome b-c1 complex subunit Rieske, mitochondrial	2.45	2
P97952	Sodium channel subunit beta-1	2.41	2
Q3TDU5	Milk fat globule-EGF factor 8 protein, isoform CRA_a	2.41	2
Q497I4	Keratin, type I cuticular Ha5	2.41	2
A2AEP2	Cyclin B3	2.40	2
P97321	Seprase	2.40	2
P97321	Seprase	2.40	2
B9EKJ4	YEATS domain containing 2	2.39	2
P61982	14-3-3 protein gamma	2.38	2
Q14B48	Coiled-coil domain-containing protein 129	2.37	2
Q14BM5	Nephrocystin-3	2.37	2
Q8R3R8	Gamma-aminobutyric acid receptor-associated protein-like 1	2.37	2
Q8VE11	Myotubularin-related protein 6	2.37	3
Q61166	Microtubule-associated protein RP/EB family member 1	2.36	5
E9QPG9	Extracellular matrix protein FRAS1	2.35	2
Q8CFE4	SCY1-like protein 2	2.35	2
O70577	Solute carrier family 22 member 2	2.30	2
B7ZNI2	Ankyrin and armadillo repeat containing	2.28	2
E9PUH2	Fam208A	2.26	2
D3Z4L1	Mthfsd	2.24	3
B7ZNX0	AF4/FMR2 family, member 4	2.23	2
Q14BE7	Family with sequence similarity 47, member A	2.23	3
Q3UW98	Chloride channel calcium activated 7	2.23	2
B6ZHD0	Erythrocyte protein band 4.1-like 2	2.23	2
E9Q8I9	Furry homolog	2.22	2
Q0V8T7	Contactin-associated protein like 5-3	2.22	2
Q9CQU1	Microfibrillar-associated protein 1	2.21	3
Q61176	Arginase-1	2.19	3
B2RQL2	Storkhead box 1	2.18	2
P27773	Protein disulfide-isomerase A3	2.18	2
E9PYY5	WD repeat-containing protein 78	2.16	2
P20065	Thymosin beta-4	2.16	2
E9QPH5	Sterile alpha motif domain-containing protein 3	2.15	2
Q9R0L7	A-kinase anchor protein 8-like	2.12	2
Q0VBL1	Tigger transposable element-derived protein 2	2.11	2
Q8VIE5	BetaIV-spectrin sigma1	2.10	2
Q08EI4	Matrix metallopeptidase 21	2.07	4

P52480	Pyruvate kinase PKM	2.04	9
Q14BE0	Molybdenum cofactor synthesis 3	2.04	3
Q61884	Meiosis-specific nuclear structural protein 1	2.04	2
Q8QZX2	HAUS augmin-like complex subunit 3	2.04	3
B9EHI6	Oviductal glycoprotein 1, 120kDa	2.03	2
Q8VDU0	G-protein signalling modulator 2	2.03	2
P30933	Seminal vesicle secretory protein 5	2.03	4
Q402U7	Serine protease 44	2.02	3
Q32NZ7	Cingulin	2.00	2
Q8C4X2	Casein kinase 1, gamma 3	2.00	3
Q9D2D7	Zinc finger protein 687	2.00	2
Q9CZP7	Hsp90 co-chaperone Cdc37-like 1	1.99	2
Q059Q0	Coiled-coil domain containing 11	1.97	3
Q8BYK4	Retinol dehydrogenase 12	1.94	3
Q91VK0	Proprotein convertase subtilisin/kexin type 5	1.93	3
B7ZMW6	CD163	1.91	2
Q3TLH4	Proline-rich coiled-coil 2C	1.91	2
Q0KIX1	KIBRA	1.91	4
Q148A4	Protein phosphatase 1 regulatory subunit 32	1.90	3
P13541	Myosin-3	1.90	2
Q9R1A8	E3 ubiquitin-protein ligase RFWD2	1.89	2
A2A9C3	Protein SZT2	1.89	2
Q5XKN4	Jagunal homolog 1	1.88	2
B7ZN50	Syntaxin binding protein 5-like	1.87	2
A0JNY7	Eukaryotic translation initiation factor 4 gamma 2	1.86	2
Q61852	Smooth muscle gamma-actin	1.86	2
D0VLQ5	Tet methylcytosine dioxygenase 2	1.85	2
P29754	Type-1A angiotensin II receptor	1.85	2
Q03142	Fibroblast growth factor receptor 4	1.85	3
Q8BFW9	Slc2a12	1.85	2
Q3UHZ5	Leiomodin-2	1.85	2
C7TQ59	Calcium channel voltage-dependent alpha1c subunit	1.83	2
Q6DFW4	Nucleolar protein 58	1.82	5
B1AR69	Myosin, heavy chain 13, skeletal muscle	1.82	2
Q01097	Glutamate receptor ionotropic, NMDA 2B	1.76	2
Q6PCN6	Microrchidia 2A	1.75	3
Q99JF5	Diphosphomevalonate decarboxylase	1.75	2
Q9DBS8	Centrosomal protein POC5	1.75	2
B2RQS6	DEAH (Asp-Glu-Ala-His) box polypeptide 36	1.74	2
Q8CJ70	Interleukin-19	1.74	2
P51881	ADP/ATP translocase 2	1.72	4
B1B1A8	Myosin light chain kinase, smooth muscle	1.72	2
Q99ML9	E3 ubiquitin-protein ligase Arkadia	1.71	3
C3S7Q5	Ojoplano variant A	1.70	2
P63028	Translationally-controlled tumor protein	1.68	2
Q8C0F9	Inactive serine protease 35	1.68	4
Q5H8C4	Vacuolar protein sorting-associated protein 13A	1.67	4
Q501L5	Osteomodulin	1.64	3
P07744	Keratin, type II cytoskeletal 4	1.63	2

Q8VII7	Receptor activity modifying protein 1	1.62	2
B9EHJ7	Tcf20 protein	1.60	2
E9QMH9	Nuclear receptor coactivator 2	1.60	2
Q8CCX5	Keratin 222	1.59	2
B7ZNV2	GLI-Kruppel family member GLI3	1.58	2
Q8C761	WD repeat-containing protein 60	1.57	3
P62082	40S ribosomal protein S7	1.56	2
B9EKS2	Jumonji domain containing 1B	1.55	2
B9EKC6	Aldehyde oxidase 3-like 1	1.54	2
Q7M759	Alpha/beta hydrolase domain-containing protein 17B	1.47	2
E9QNG9	Serine/threonine-protein kinase B-raf	1.44	2
P08730	Keratin, type I cytoskeletal 13	1.39	2
B7ZND9	Testis expressed gene 9	1.38	2
Q8K224	N-acetyltransferase 10	1.37	2
P17751	Triosephosphate isomerase	1.36	2
E9Q401	Ryanodine receptor 2	1.35	5
Q05D94	Copine VIII	1.35	2
Q0KK56	Family with sequence similarity 184, member B	1.33	4
Q3TRR0	Microtubule-associated protein 9	1.33	2
P53349	Mitogen-activated protein kinase kinase kinase 1	1.29	9
P70452	Syntaxin-4	1.28	2
P61082	NEDD8-conjugating enzyme Ubc12	1.27	2
Q7TQG5	Neogenin	1.27	2
Q3UEH4	Pyruvate kinase liver and red blood cell	1.26	2
E9Q555	E3 ubiquitin-protein ligase RNF213	1.17	5
Q8VG97	Olfactory receptor 1132	1.16	3
Q8VG50	Olfactory receptor 922	1.15	2
Q62018	RNA polymerase-associated protein CTR9 homolog	1.14	2
Q8CHW3	Mitochondrial ribosomal protein L23	1.11	3
Q9JKE5	ELKL motif kinase 2 long form	1.05	2
Q99JY8	Lipid phosphate phosphohydrolase 3	1.04	2
Q9D168	Integrator complex subunit 12	0.96	2
Q61784	ORF1	0.91	2
C9K0Z6	CNTF receptor alpha subunit	0.89	2
Q80UH6	Nucleotide-binding oligomerization domain containing 2	0.89	2
Q0VG46	Calcyphosphine 2	0.85	2
Q7TSF0	Desmoglein-1-gamma	0.84	2
B7ZWF1	DEAD (Asp-Glu-Ala-Asp) box helicase 3, X-linked	0.71	2
Q2YFS4	Zinc finger CW-type PWWP domain protein 1	0.34	2
